# Supplementary material for: A biotin targeting chimera (BioTAC) system to map small molecule interactomes in situ
Source: Nat Commun. 2023 Dec 4;14:8016. doi: 10.1038/s41467-023-43507-5 (PMC10695998; doi:10.1038/s41467-023-43507-5)
Supplement: Supplementary file 1 — Supplementary Information [file 41467_2023_43507_MOESM1_ESM.pdf]

A Biotin Targeting Chimera (BioTAC) System to Map Small Molecule Interactomes *in situ*

Andrew J. Tao[*,1], Jiewei Jiang[*,1], Gillian E. Gadbois[1], Pavitra Goyal[1], Bridget T. Boyle[1], Elizabeth J. Mumby[2], Samuel A Myers[3] Justin G. English[2,#], Fleur M. Ferguson[1,4,#]

[1]Department of Chemistry and Biochemistry, University of California San Diego, La Jolla, CA 92093.

[2]Department of Biochemistry, University of Utah School of Medicine, Salt Lake City, UT 84112.

[3] Laboratory for Immunochemical Circuits, La Jolla Institute for Immunology, La Jolla, CA 92037

[4]Skaggs School of Pharmacy and Pharmaceutical Sciences, University of California San Diego, La Jolla, CA 92093.

*These authors contributed equally.

[#]Correspondance: justin.english@biochem.utah.edu, fmferguson@ucsd.edu.

Contents

Supplementary Figure 1 | Optimization of (+)-JQ1 orthoAP1867 bifunctional molecules…………2

Supplementary Figure 2 | Colocalization of BioTAC constructs and BRD4…………………………3

Supplementary Figure 3 | Comparison of the BioTAC system to photoaffinity labeling and μMap..4

Supplementary Figure 4 | Volcano plots of proteomic experiments…………………………………5

Supplementary Figure 5 | Optimization of alternate-linker (+)-JQ1 orthoAP1867 bifunctional molecules……………………………………………………………………………………………7

Supplementary Figure 6 | Optimization of alisertib-orthoAP1867 bifunctional molecules…………8

Supplementary Figure 7 | Optimization of MEK1 labeling by trametinib and trametiglue bifunctional molecules………………………………………………………………………………9

Supplementary Figure 8 | Colocalization of BioTAC constructs and MEK1/2……………………...11

Supplementary Figure 9 | Optimization of the BioTAC system……………………………………...12

Chemistry Methods…………………………………………………………………………………...13

Supplementary References……………………………………………………………………………31

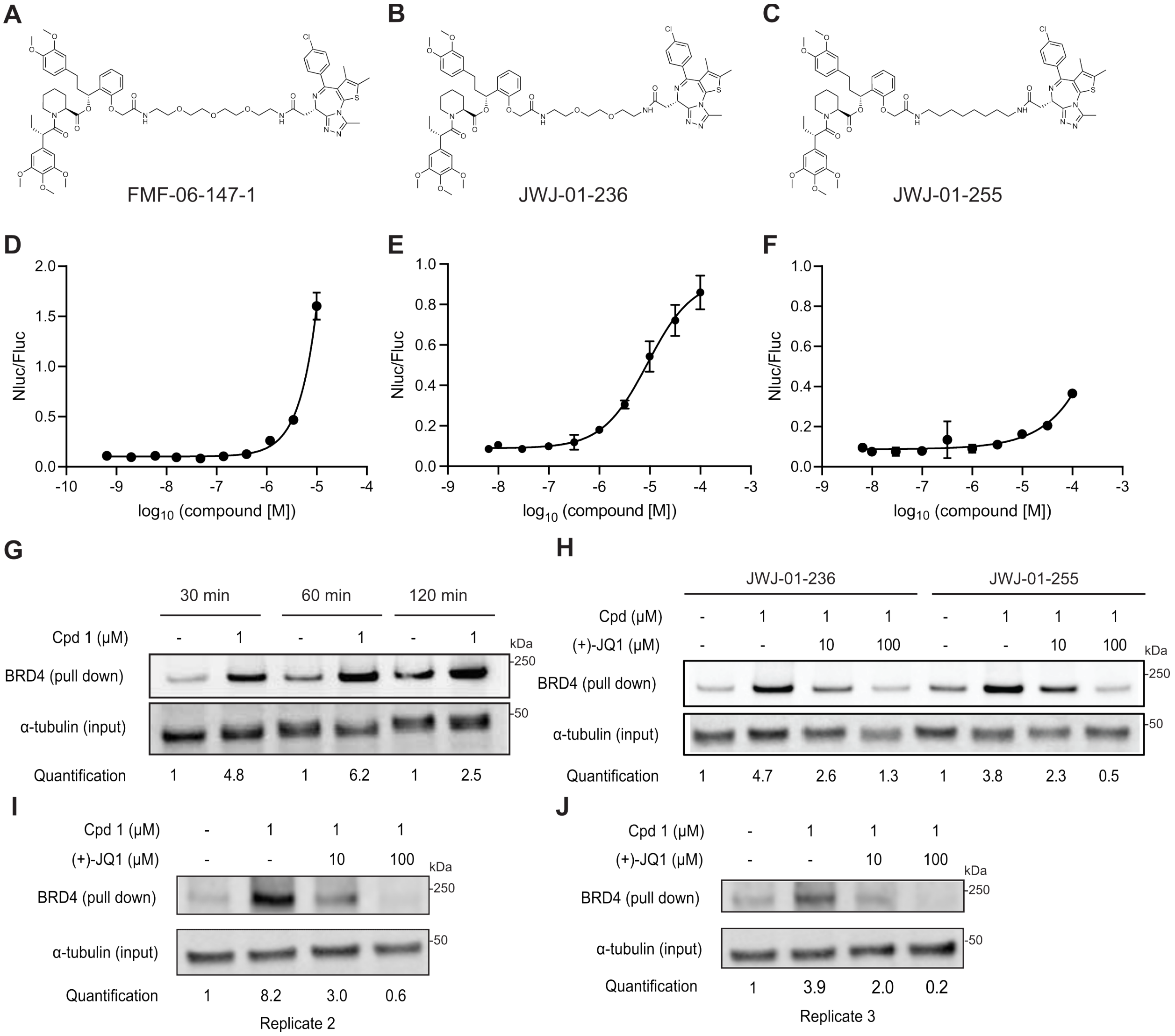

Supplementary Figure 1 | Optimization of (+)-JQ1 orthoAP1867 bifunctional molecules. A.-C. Chemical structure of (+)-JQ1 bifunctional molecule analogs. D.-F. FKBP12^{F36V} cellular target engagement assays for compounds shown A.-C., data plotted as mean ± SD of *n* = 3 technical replicates. D. FMF-06-147-1, E. JWJ-01-236, F. JWJ-01-255 demonstrating competitive displacement of dTAG-13. G. Immunoblot analysis of BRD4 enrichment following treatment of HEK293 cells transiently transfected with miniTurboFKBP12^{F36V} and treated with FMF-06-147-1 and 100 μM biotin at the indicated timepoints (input = sample processing control, *n* = 2 biological replicates). H. Immunoblot analysis of BRD4 enrichment following treatment of HEK293 cells transiently transfected with miniTurboFKBP12^{F36V} and treated with the indicated compounds and 100 μM biotin at the 60 min timepoint (input = sample processing control, *n* = 2 biological replicates). I., J. Biological replicates of Figure 1C, immunoblot analysis of BRD4 enrichment following treatment of HEK293 cells transiently transfected with miniTurboFKBP12^{F36V} and treated with FMF-06-147-1 and 100 μM biotin at the 30 min timepoint (input = sample processing control). SD standard deviation. Source data are provided as a Source data file.

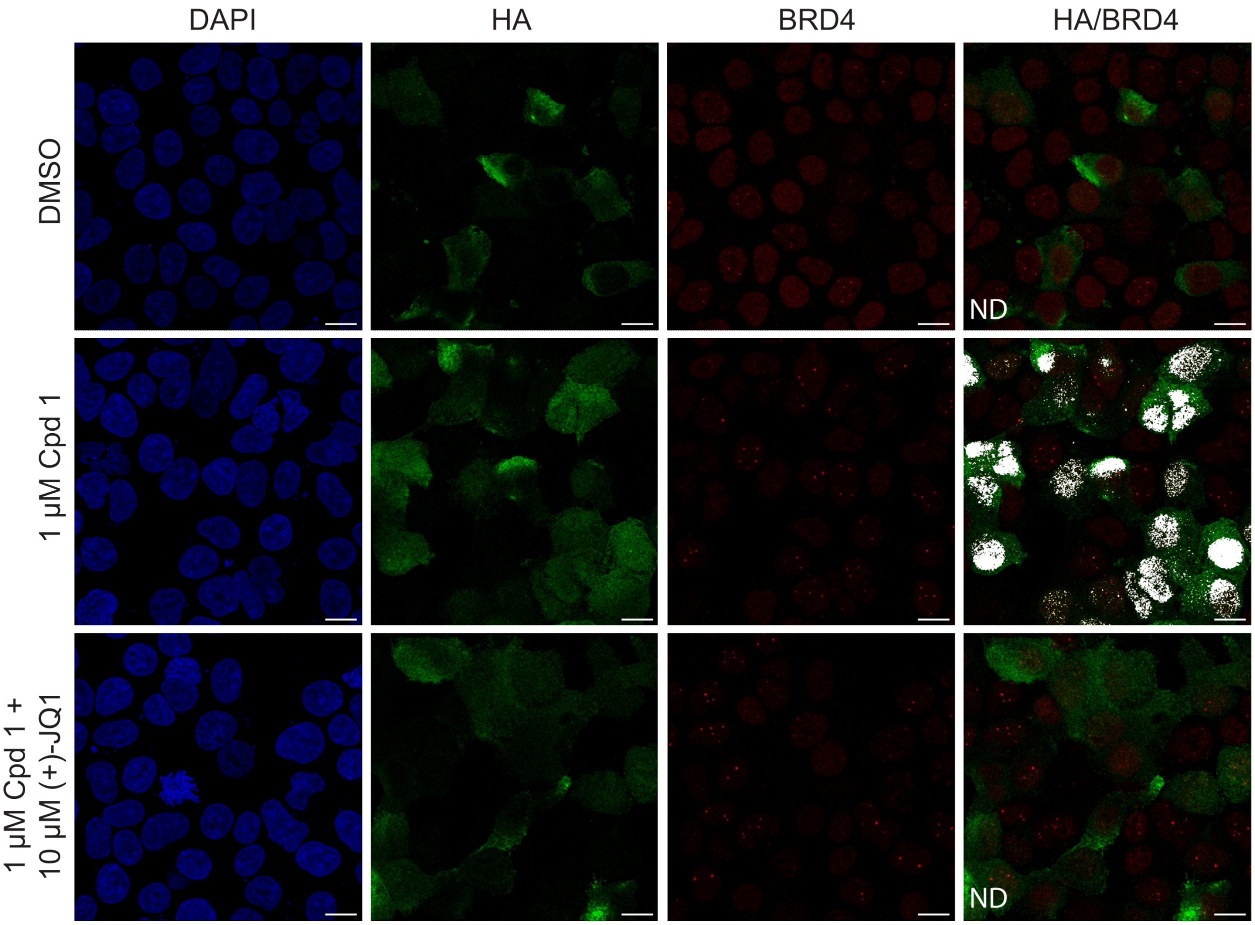

Supplementary Figure 2 | Colocalization of BioTAC constructs and BRD4. Representative maximum intensity projections of confocal microscopy images of HEK293 cells transiently transfected with miniTurboFKBP12^{F36V} and treated with the indicated compounds for 30 min. Blue: DAPI (nucleus), green: HA (miniTurboFKBP12^{F36V}), red: BRD4, white: colocalization, ND: no colocalization detected. 10 Z-stacks were collected per image and processed using colocalization thresholding between HA and BRD4. Scale bar = 10 µm, *n* = 2 technical replicates.

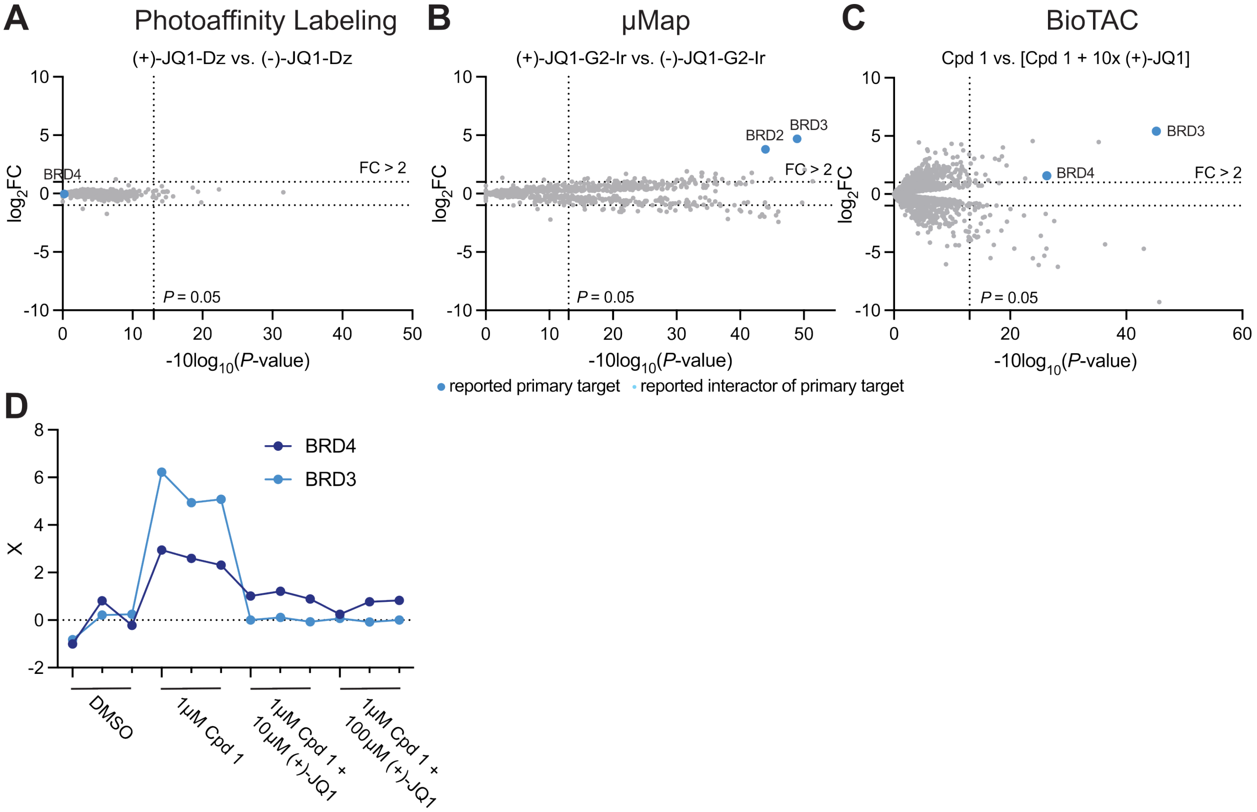

Supplementary Figure 3 | Comparison of the BioTAC system to photoaffinity labeling and μMap. A.-B. Replotted reference data from Trowbridge *et. al.* showing photoaffinity labeling and μMap results for (+)-JQ1. C. Proteomics analysis of biotinylated proteins enriched by streptavidin pulldown from HEK293 cells transiently transfected with miniTurbo-FKBP12^{F36V} and treated with 100 μM Biotin, 1 μM of Cpd 1 ± 10 μM (+)-JQ1 for 60 min. D. F-test analysis of proteomic data depicted in C., showing significant enrichment of BRD3 and BRD4 in the presence of 1 μM Cpd 1, relative to DMSO and (+)-JQ1 off-compete experiments. X scaled abundance ratio. FC fold-change, complete datasets in Supplementary Dataset 1. Source data are provided as a Source Data file.

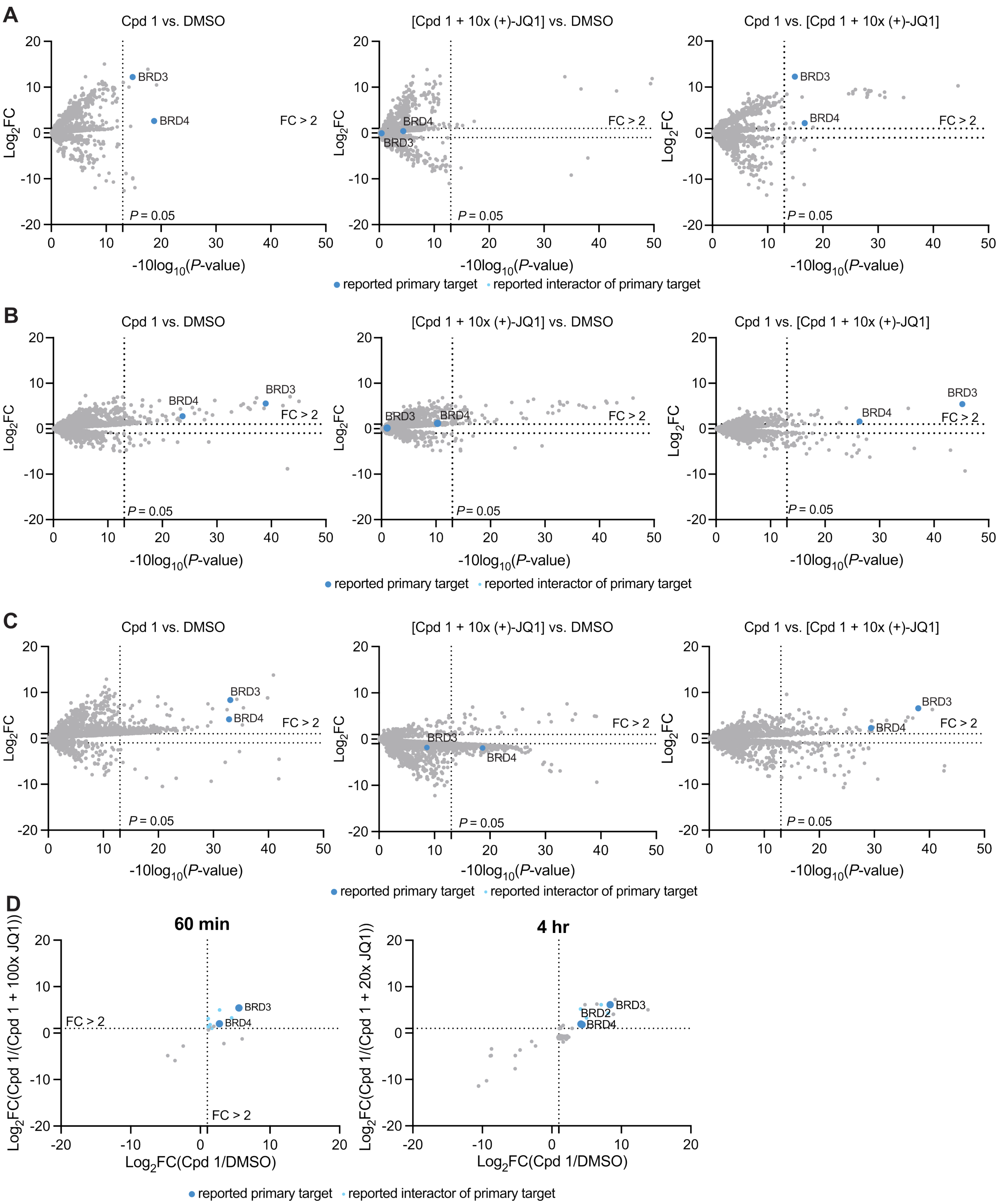

Supplementary Figure 4 | Volcano plots of proteomic experiments. A.-C. Volcano plots showing all datapoints from mass-spectrometry based proteomic analysis of proteins enriched by streptavidin pulldown from HEK293 cells transiently transfected with miniTurbo-FKBP12^{F36V} and treated with 100 μM Biotin, DMSO or 1 μM of Cpd 1 ± 10 μM (+)-JQ1 for A. 30 min, B. 60 min, C. 4 hr. Corresponding to Figure 2A. D. Time-course proteomics of streptavidin-enriched biotinylated proteins isolated from HEK293 cells transiently transfected with

miniTurboFKBP12^{F36V} and treated with the indicated compounds and 100 μM biotin, demonstrating enrichment and competition of known direct targets and complexed proteins (60 min, 4 hr). Only proteins with *P*-value < 0.05 in both conditions depicted. High-confidence hits are defined as those that are enriched > 2-fold in both Cpd 1 / DMSO and Cpd 1 / Cpd 1 + 20x or 100x (+)-JQ1, where *P* < 0.05 (two-sample moderated T test), plotted upper-right quadrant. FC fold-change, complete datasets in Supplementary Dataset 1. Source data are provided as a Source Data file.

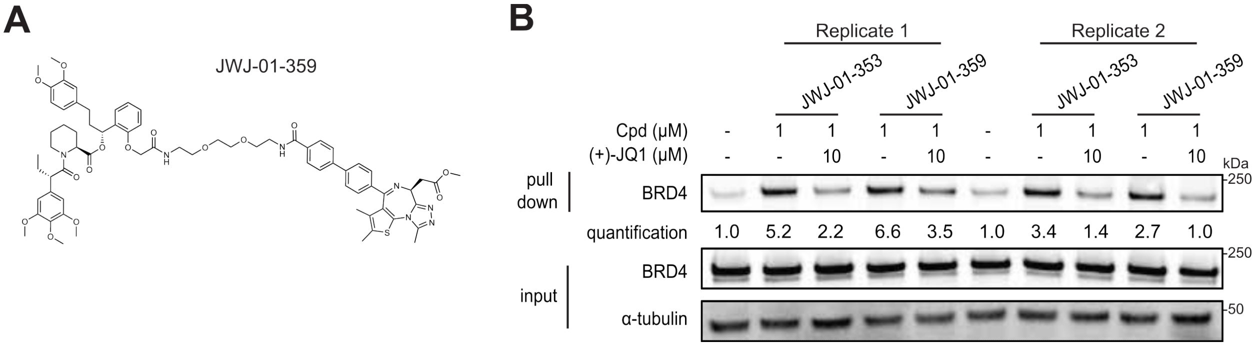

Supplementary Figure 5 | Optimization of alternate-linker (+)-JQ1 orthoAP1867 bifunctional molecules. A. Chemical structure of JWJ-01-359. B. Immunoblot analysis of BRD4 enrichment following treatment of HEK293 cells transiently transfected with miniTurboFKBP12^{F36V} and treated with the indicated compound and 100 μM biotin at the 30 min timepoint (input = sample processing control, *n* = 2 biological replicates). Source data are provided as a Source Data file.

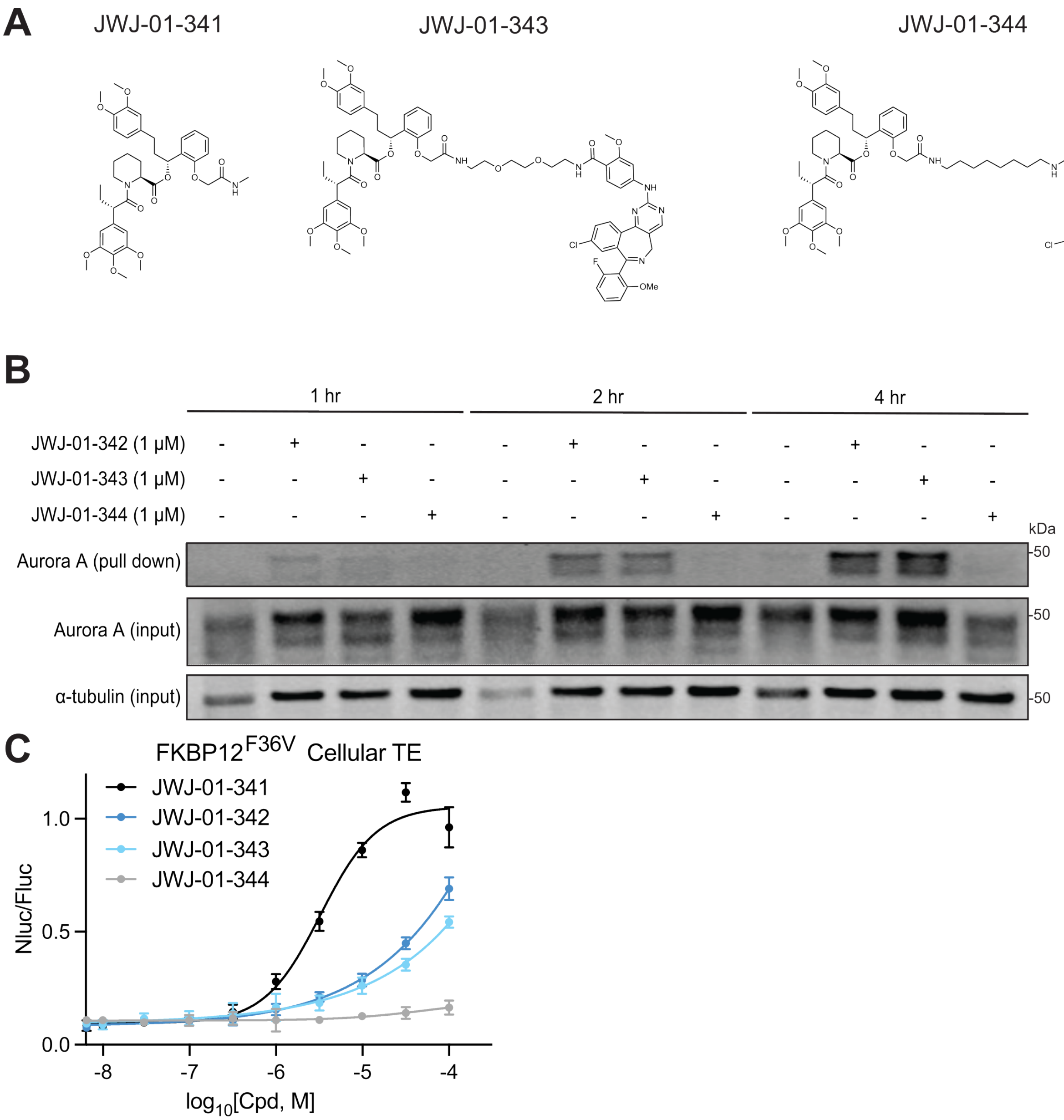

Supplementary Figure 6 | Optimization of alisertib-orthoAP1867 bifunctional molecules. A. Chemical structure of control compound JWJ-01-341 and alisertib bifunctional molecule analogs. B. Immunoblot analysis of Aurora A enrichment following treatment of HEK293 cells transiently transfected with miniTurboFKBP12^{F36V} and treated with the indicated compound and 100 μM biotin at the indicated timepoint (input = sample processing control, *n* = 2 biological replicates). C. FKBP12^{F36V} cellular target engagement assays for alisertib bifunctional molecules, data plotted as mean ± SD of *n* = 3 technical replicates. SD standard deviation. Source data are provided as a Source Data file.

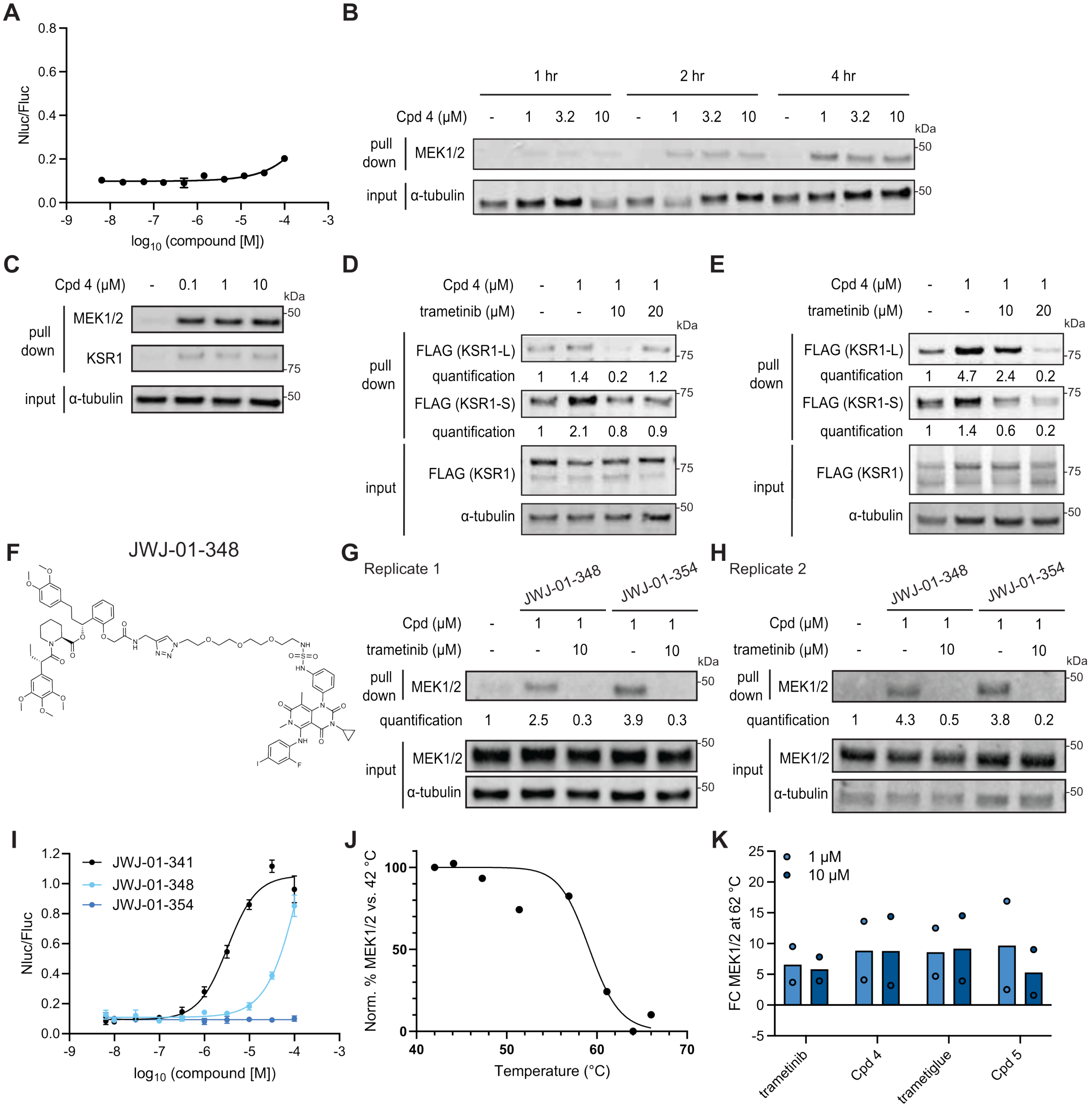

Supplementary Figure 7 | Optimization of MEK1 labeling by trametinib and trametiglue bifunctional molecules. A. FKBP12^{F36V} cellular target engagement assays for JWJ-01-280, data plotted as mean ± SD of *n* = 3 technical replicates. B. Immunoblot analysis of MEK1/2 enrichment following treatment of HEK293 cells transiently transfected with miniTurboFKBP12^{F36V} with JWJ-01-280 and 100 μM biotin at the indicated timepoint (*n* = 2 biological replicates). C. Immunoblot analysis of MEK1/2 and KSR1 enrichment following treatment of HEK293 cells transiently transfected with miniTurboFKBP12^{F36V} with the indicated concentration of JWJ-01-280 and 100 μM biotin for 4 hr (*n* = 2 biological replicates). D., E. Biological replicates of Figure 5D, immunoblot analysis of mouse KSR1 following treatment of HEK293 cells transiently transfected with miniTurbo-FKBP12F36V and mKSR1, with the indicated compounds and 100 μM biotin at the 4 hr timepoint, and streptavidin-based enrichment, showing successful enrichment and competition with trametinib (input = sample processing control). In D., E. two KSR1 isoforms are observed, produced by alternative splicing. KSR1-L (102 KDa) corresponds to the expected molecular

weight of Uniprot Q8IVT5-1 (canonical sequence), KSR1-S (87 KDa) corresponds to the expected molecular weight of variant with residues 1-137 missing, Uniprot Q8IVT5-3, and -4. Our data indicate both isoforms can complex with trametinib-bound MEK1. F. Chemical structure of JWJ-01-348. G., H. Technical replicates of immunoblot analysis of MEK1/2 and KSR1 enrichment following treatment of HEK293 cells transiently transfected with miniTurboFKBP12^{F36V} with the indicated concentration of the indicated compound and 100 μM biotin for 4 hr (input = sample processing control). I. FKBP12^{F36V} cellular target engagement assays for trametiglue bifunctional molecules, data plotted as mean ± SD of *n* = 3 technical replicates. J. CETSA analysis of MEK1/2 melting point. Data based on western blot quantification, normalized to the lowest temperature of 42 °C (*n* = 1 biological replicate). K. Thermal stabilization of MEK1/2 by inhibitors and bifunctional derivatives (*n* = 2 biological replicates). HEK293 cells were treated with the indicated compounds at the indicated concentrations for 3 min, followed by isolation and incubation at 62 °C. Data based on western blot quantification, normalized to DMSO treated controls. SD Standard deviation. Source data are provided as a Source Data file.

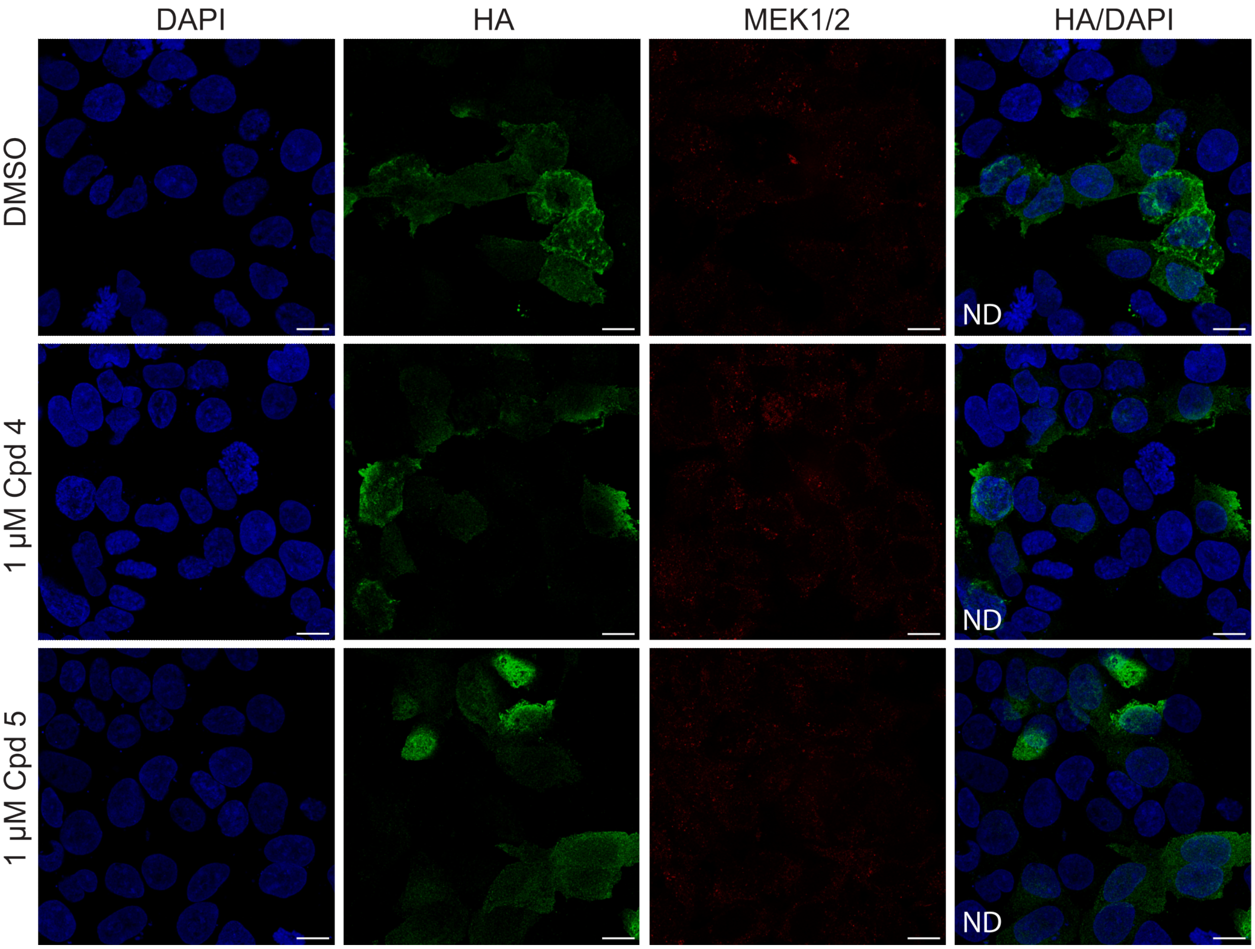

Supplementary Figure 8 | Colocalization of BioTAC constructs and MEK1/2. Representative maximum intensity projections of confocal microscopy images of HEK293 cells transiently transfected with miniTurboFKBP12^{F36V} and treated with the indicated compounds for 4 hr. Blue: DAPI (nucleus), green: HA (miniTurboFKBP12^{F36V}), red: MEK1/2, white: colocalization, ND: no correlation detected. 10 Z-stacks were collected per image and processed using colocalization thresholding between HA and DAPI. Scale bar = 10 µm, *n* = 2 technical replicates.

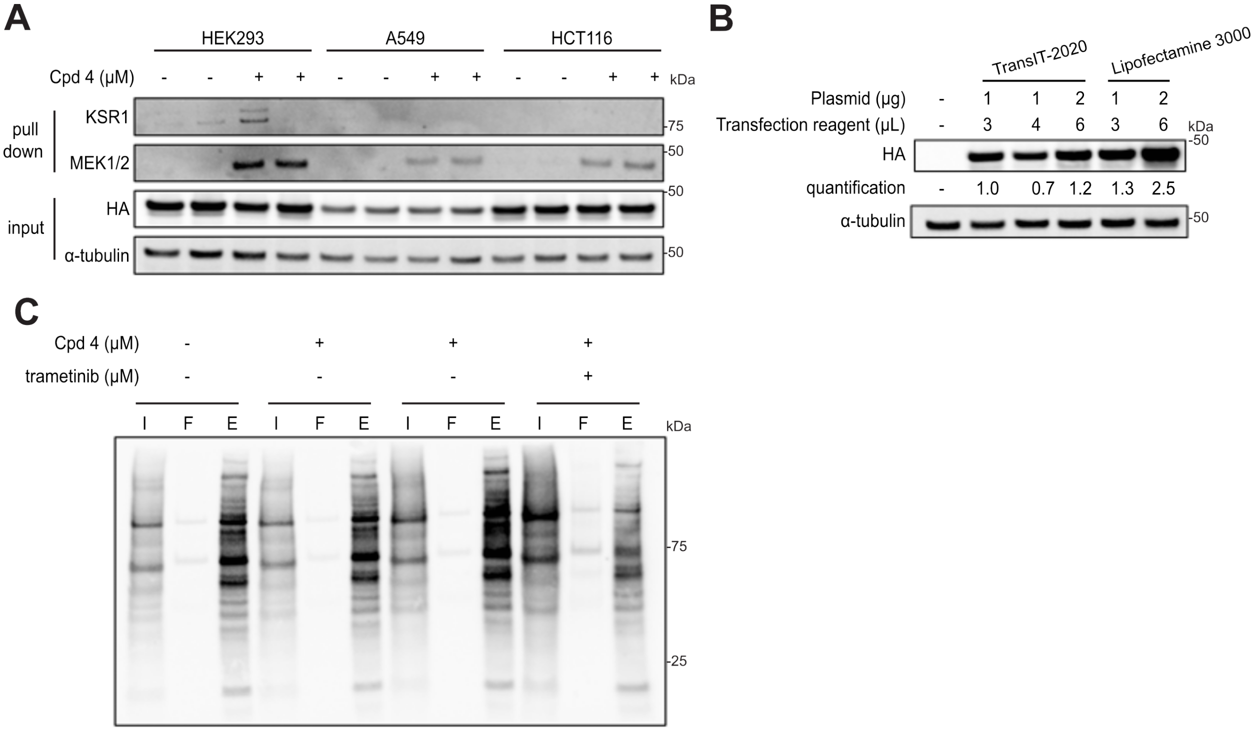

Supplementary Figure 9 | Optimization of the BioTAC system. A. Immunoblot analysis of MEK1/2 and KSR1 enrichment following treatment of indicated cell lines transiently transfected with miniTurboFKBP12^{F36V} with 1 μM Cpd 4 and 100 μM biotin for 4 hr (input = sample processing control, *n* = 1 biological replicate). B. Immunoblot analysis of BioTAC construct transfection efficiency in A549 cells transiently transfected with miniTurboFKBP12F36V under the indicated conditions (*n* = 1 biological replicate). C. Immunoblot analysis of global biotinylation in input (I), flowthrough (F), and enrichment (E) samples following treatment of HEK293 cells transiently transfected with miniTurboFKBP12^{F36V} with 1 μM Cpd 4 and 100 μM biotin for 4 hr (*n* = 1 biological replicate). Source data are provided as a Source Data file.

CHEMISTRY METHODS

General Methods

Unless otherwise noted, reagents and solvents were obtained from commercial suppliers and were used without further purification. ^{1}H NMR spectra were recorded on 500 MHz Bruker Avance III spectrometer or 500 MHz JEOL ECA 500 spectrometer and chemical shifts are reported in parts per million (ppm, δ) downfield from tetramethylsilane (TMS). Coupling constants (J) are reported in Hz. Spin multiplicities are described as s (singlet), br (broad singlet), d (doublet), t (triplet), q (quartet), and m (multiplet). Mass spectra were obtained on a Waters Acquity UPLC/MS. Preparative HPLC was performed on a Waters Sunfire C18 column (19 mm × 50 mm, 5 μM) using a gradient of 15−95% methanol in water containing 0.05% trifluoroacetic acid (TFA) over 22 min (28 min run time) at a flow rate of 20 mL/min. Assayed compounds were isolated and tested as TFA salts. Purities of assayed compounds were in all cases greater than 95%, as determined by reverse-phase HPLC analysis.

tert-Butyl (S)-(1-(4-(4-chlorophenyl)-2,3,9-trimethyl-6H-thieno[3,2-f][1,2,4]triazolo[4,3-a][1,4]diazepin-6-yl)-2-oxo-6,9,12-trioxa-3-azatetradecan-14-yl)carbamate (2)

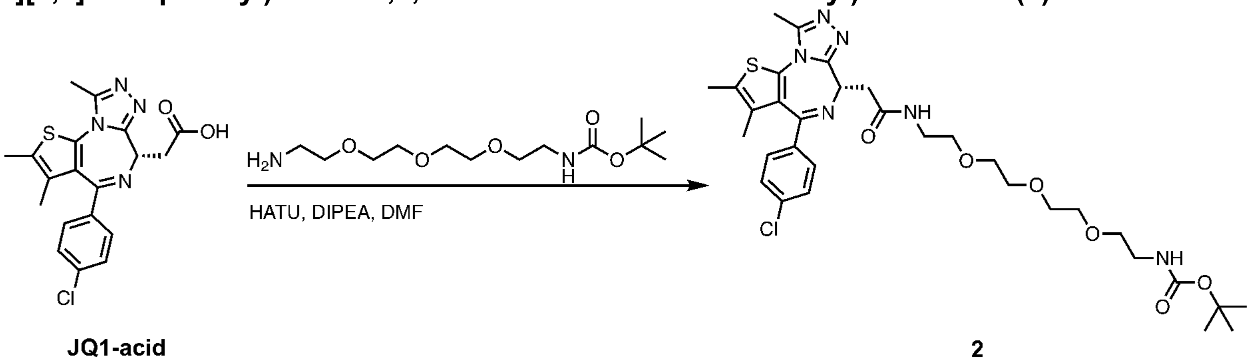

JQ1-acid **2**

JQ1-acid (25 mg, 0.06 mmol), *tert*-butyl (2-(2-(2-(2-aminoethoxy)ethoxy)ethoxy)ethyl)carbamate (20 mg, 0.066 mmol), HATU (28 mg, 0.07 mmol), DIPEA (35 μL, 0.18 mmol) were dissolved in DMF (2 mL) and stirred at rt for 12 h. The reaction mixture was diluted in conc. aq. sodium bicarbonate (10 mL) and extracted with DCM (3 x 10 mL). The organics were combined, dried over $MgSO_4$, filtered and concentrated *in vacuo*. The crude product was purified via flash column chromatography (DCM:MeOH) to yield the title compound (27 mg, 0.04 mmol, 63%) as a colorless oil. MS (ESI) m/z 676 $(M + H)^+$.

(*S*)-*N*-(2-(2-(2-(2-aminoethoxy)ethoxy)ethoxy)ethyl)-2-(4-(4-chlorophenyl)-2,3,9-trimethyl-6*H*-thieno[3,2-*f*][1,2,4]triazolo[4,3-*a*][1,4]diazepin-6-yl)acetamide (3)

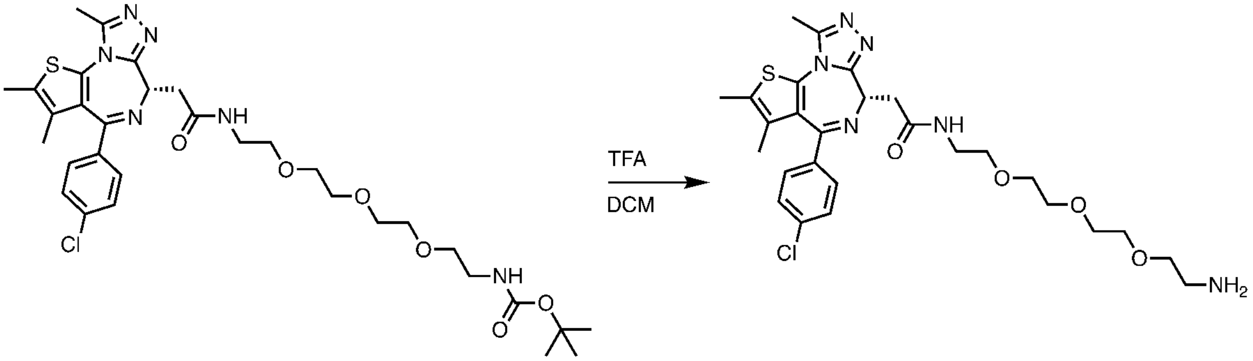

2 **3**

Compound 2 (27 mg, 0.04 mmol) was dissolved in 2 mL DCM and 0.5 mL TFA, and stirred at rt for 2 h. The reaction mixture was concentrated *in vacuo* and used without further purification (quant.). MS (ESI) m/z 576 $(M + H)^+$.

(*R*)-1-(2-((17-((*S*)-4-(4-Chlorophenyl)-2,3,9-trimethyl-6*H*-thieno[3,2-*f*][1,2,4]triazolo[4,3-*a*][1,4]diazepin-6-yl)-2,16-dioxo-6,9,12-trioxa-3,15-diazaheptadecyl)oxy)phenyl)-3-(3,4-dimethoxyphenyl)propyl (*S*)-1-((*S*)-2-(3,4,5-trimethoxyphenyl)butanoyl)piperidine-2-carboxylate (FM-06-147-1)

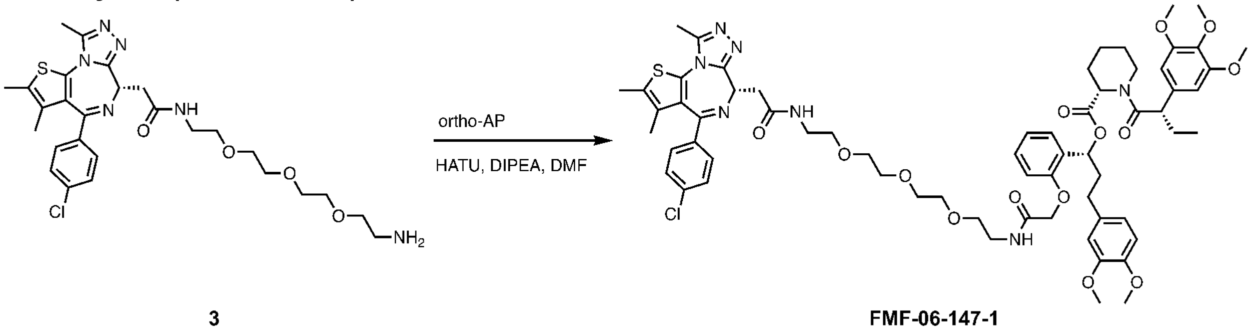

3 **FMF-06-147-1**

Compound 3 (25 mg, 0.04 mmol), ortho-AP (28 mg, 0.04 mmol), HATU (19 mg, 0.05 mmol), DIPEA (22 µL, 0.12 mmol) were dissolved in DMF (2 mL) and stirred at rt for 16 h. The reaction mixture was filtered and purified by HPLC to afford the title compound as a TFA salt (24 mg, 0.017 mmol), as a white solid. MS (ESI) m/z 626 $(M + 2H)^{2+}/2$. ^1H NMR (400 MHz, DMSO-d_6) δ 8.31 (t, *J* = 5.7 Hz, 1H), 7.85 (t, *J* = 5.7 Hz, 1H), 7.48 (d, *J* = 7.8 Hz, 2H), 7.42 (d, *J* = 8.6 Hz, 2H), 7.19 (ddd, *J* = 8.6, 6.6, 2.5 Hz, 1H), 6.88–6.83 (m, 1H), 6.83–6.76 (m, 3H), 6.74 (d, *J* = 2.0 Hz, 1H), 6.67–6.60 (m, 1H), 6.55 (s, 2H), 6.02 (dd, *J* = 8.3, 4.7 Hz, 1H), 5.33 (dd, *J* = 6.0, 2.5 Hz, 1H), 4.59 (d, *J* = 7.8 Hz, 1H), 4.56–4.48 (m, 3H), 4.05 (d, *J* = 13.4 Hz, 1H), 3.87 (dd, *J* = 8.1, 6.1 Hz, 1H), 3.74–3.68 (m, 8H), 3.64 (d, *J* = 10.6 Hz, 1H), 3.54 (d, *J* = 6.6 Hz, 8H), 3.49 (d, *J* = 5.1 Hz, 4H), 3.48–3.38 (m, 8H), 3.32–3.17 (m, 6H), 2.61 (s, 4H), 2.40 (s, 4H), 2.16 (d, *J* = 13.4 Hz, 1H), 2.08–1.83 (m, 2H), 1.66–1.49 (m, 7H), 1.42–1.07 (m, 1H), 0.76 (dt, *J* = 29.1, 7.2 Hz, 3H). ^{13}C NMR (100 MHz, DMSO-D_6) δ 172.53, 170.96, 170.12, 168.11, 163.80, 154.59, 153.37, 153.19, 149.10, 147.50, 137.06, 136.76, 136.46, 136.11, 135.91, 133.84, 132.68, 131.56, 130.78, 130.45, 130.18, 129.35, 129.16, 129.00, 126.51, 121.66, 120.46, 112.61, 112.26, 105.52, 105.28, 70.27, 70.21, 70.11, 70.08, 69.72, 69.35, 67.63, 60.52, 60.28, 56.39, 55.98, 55.95, 55.78, 54.22, 51.95, 49.22, 43.43, 39.14, 38.79, 37.85, 36.93, 31.08, 28.61, 26.81, 25.48, 21.02, 14.56, 13.18, 12.88, 12.79, 11.80. HRMS (m/z): $[M+H]^+$ calcd. for $C_{65}H_{81}ClN_7O_{14}S$, 1250.5245; found, 1250.5236.

***tert*-Butyl (*S*)-(8-(2-(4-(4-chlorophenyl)-2,3,9-trimethyl-6*H*-thieno[3,2-*f*][1,2,4]triazolo[4,3-*a*][1,4]diazepin-6-yl)acetamido)octyl)carbamate (4)**

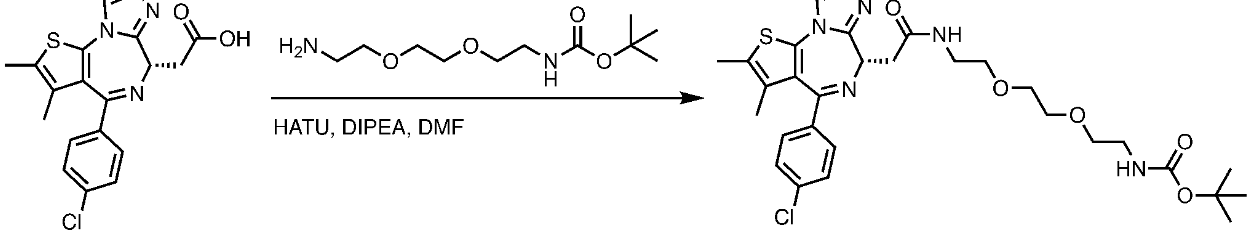

JQ1-acid **4**

JQ1-acid (25 mg, 0.062 mmol), HATU (28 mg, 0.075 mmol), DIPEA (33 µL, 0.19 mmol), and *tert*-butyl (2-(2-(2-aminoethoxy)ethoxy)ethyl)carbamate (17 mg, 0.069 mmol) were charged into a 4 mL vial. After adding 1 mL DMF, the solution was stirred for 2 hours. Upon the completion of the coupling reaction indicated by LCMS, the material was partitioned between DCM (15 mL) and

brine (15 mL), and transferred to a separatory funnel. The layers were separated, and the aqueous phase was further extracted with DCM (15 mL). The organic extracts were dried over $MgSO_4$, filtered, and concentrated to dryness. The residue was purified by silica gel chromatography to yield 37 mg (95%) of the title compound. ^{1}H NMR (500 MHz, Methanol-*d*$_4$) δ 7.44 (d, *J* = 8.3 Hz, 2H), 7.39 (d, *J* = 8.7 Hz, 2H), 4.61 (dd, *J* = 8.9, 5.3 Hz, 1H), 3.61 (ddd, *J* = 11.9, 6.9, 3.5 Hz, 6H), 3.50 (t, *J* = 5.7 Hz, 2H), 3.46–3.42 (m, 2H), 3.31 (d, *J* = 5.2 Hz, 1H), 3.27 (s, 2H), 3.20 (t, *J* = 5.6 Hz, 2H), 2.68 (s, 3H), 2.43 (s, 3H), 1.68 (s, 3H), 1.40 (s, 9H), 1.29 (dd, *J* = 12.4, 6.3 Hz, 1H). MS (ESI) m/z 631.4 $(M + H)^+$.

(*S*)-*N*-(2-(2-(2-Aminoethoxy)ethoxy)ethyl)-2-(4-(4-chlorophenyl)-2,3,9-trimethyl-6*H*-thieno[3,2-*f*][1,2,4]triazolo[4,3-*a*][1,4]diazepin-6-yl)acetamide (5)

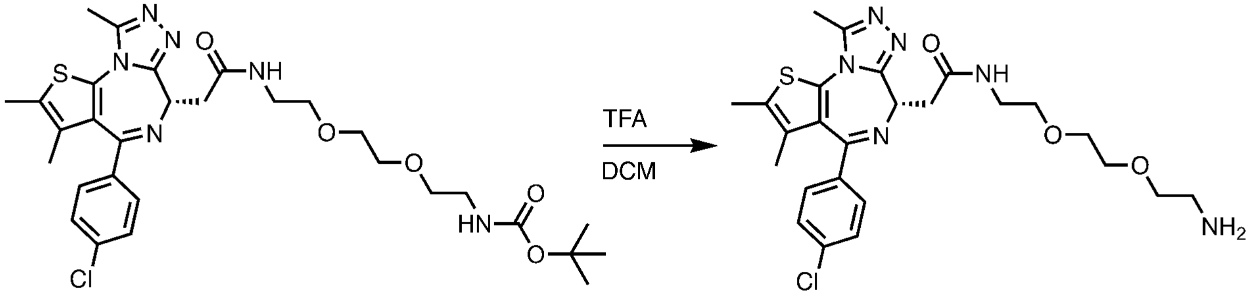

4 **5**

Compound 4 (37 mg, 0.06 mmol) was dissolved in 2 mL DCM and 0.5 mL TFA, and stirred at rt for 2 h. The reaction mixture was concentrated *in vacuo* and used without further purification (quant.). MS (ESI) m/z 531.4 $(M + H)^+$.

(*R*)-1-(2-((14-((*S*)-4-(4-Chlorophenyl)-2,3,9-trimethyl-6*H*-thieno[3,2-*f*][1,2,4]triazolo[4,3-*a*][1,4]diazepin-6-yl)-2,13-dioxo-6,9-dioxa-3,12-diazatetradecyl)oxy)phenyl)-3-(3,4-dimethoxyphenyl)propyl (*S*)-1-((*S*)-2-(3,4,5-trimethoxyphenyl)butanoyl)piperidine-2-carboxylate (JWJ-01-236)

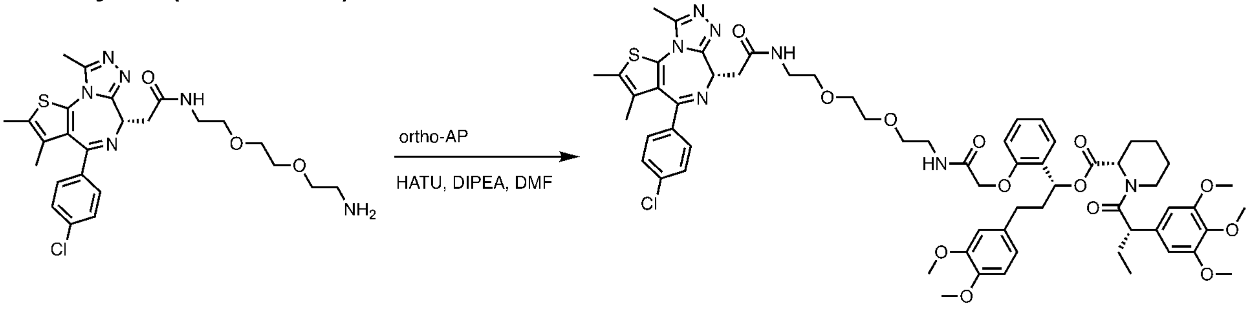

5 **JWJ-01-236**

Compound 5 (37 mg, 0.05 mmol), ortho-AP (36 mg, 0.05 mmol), HATU (22 mg, 0.06 mmol), DIPEA (25 µL, 0.14 mmol) were dissolved in DMF (2 mL) and stirred at rt for 16 h. The reaction mixture was filtered and purified by HPLC to afford the title compound as a while solid (1.6 mg, 2.6%) with 95% purity. ^{1}H NMR (500 MHz, DMSO-*d*$_6$) δ 8.27 (s, 1H), 7.85 (t, *J* = 5.7 Hz, 1H), 7.47 (d, *J* = 8.7 Hz, 2H), 7.41 (d, *J* = 8.2 Hz, 2H), 7.19 (ddt, *J* = 8.7, 6.5, 3.3 Hz, 1H), 6.88–6.76 (m, 4H), 6.74 (d, *J* = 2.0 Hz, 1H), 6.66–6.60 (m, 2H), 6.55 (s, 2H), 6.02 (dd, *J* = 8.3, 4.7 Hz, 1H), 5.32 (t, *J* = 4.2 Hz, 1H), 4.50 (q, *J* = 5.0 Hz, 3H), 4.06–4.03 (m, 2H), 3.87 (s, 2H), 3.71 (s, 3H), 3.52–3.47 (m, 6H), 3.42 (q, *J* = 6.1 Hz, 5H), 3.26 (tdd, *J* = 16.4, 12.6, 8.8 Hz, 8H), 2.58 (s, 6H), 2.40 (s, 4H), 2.15 (d, *J* = 14.0 Hz, 1H), 2.02–1.83 (m, 3H), 1.66–1.50 (m, 8H), 1.31–1.20 (m, 4H), 0.76 (dt, *J* = 45.9, 7.3 Hz, 3H). ^{13}C NMR (100 MHz, DMSO-*D*$_6$) δ 172.54, 170.97, 170.16, 168.13, 163.74, 154.60, 153.37, 153.20, 149.10, 147.50, 137.12, 136.77, 136.46, 136.11, 135.87, 133.85,

132.72, 131.46, 130.75, 130.43, 130.15, 129.35, 129.16, 128.99, 126.51, 121.66, 120.46, 112.61, 112.25, 105.52, 105.28, 70.36, 70.06, 69.72, 69.37, 67.64, 60.51, 60.28, 56.39, 55.98, 55.95, 55.77, 54.27, 51.95, 49.22, 39.13, 38.80, 37.90, 36.93, 31.09, 28.62, 28.25, 26.81, 25.50, 21.04, 14.55, 13.17, 12.89, 12.79, 11.80. MS (ESI) *m/z* 1206.6 $(M + H)^+$.

***tert*-Butyl (*S*)-(8-(2-(4-(4-chlorophenyl)-2,3,9-trimethyl-6*H*-thieno[3,2-*f*][1,2,4]triazolo[4,3-*a*][1,4]diazepin-6-yl)acetamido)octyl)carbamate (6)**

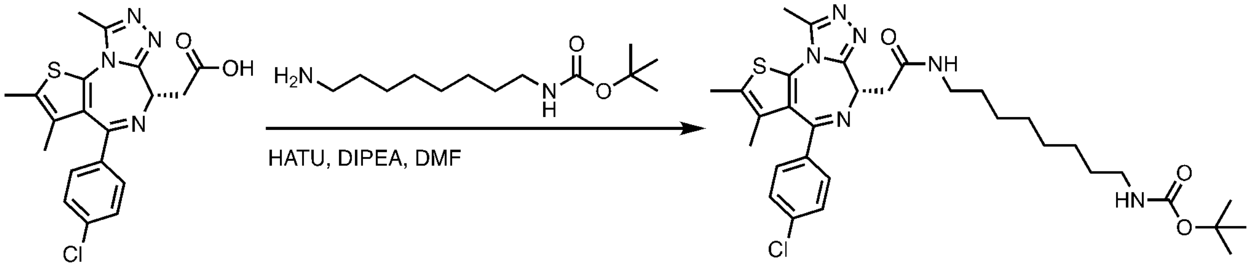

JQ1-acid **6**

JQ1-acid (15 mg, 0.037 mmol), HATU (14 mg, 0.056 mmol), DIPEA (20 μL, 0.11 mmol), and *tert*-butyl (8-aminooctyl)carbamate (11 mg, 0.045 mmol) were charged into a 4 mL vial. After adding 1 mL DMF, the solution was stirred for 2 hours. Upon the completion of the coupling reaction indicated by LCMS, the material was partitioned between DCM (15 mL) and brine (15 mL), and transferred to a separatory funnel. The layers were separated, and the aqueous phase was further extracted with DCM (15 mL). The organic extracts were dried over magnesium sulfate, filtered, and concentrated to dryness. The residue was purified by silica gel chromatography to yield 23 mg (99%) of the title compound, MS (ESI) m/z 627.4 $(M + H)^+$.

(*S*)-*N*-(8-Aminooctyl)-2-(4-(4-chlorophenyl)-2,3,9-trimethyl-6*H*-thieno[3,2-*f*][1,2,4]triazolo[4,3-*a*][1,4]diazepin-6-yl)acetamide (7)

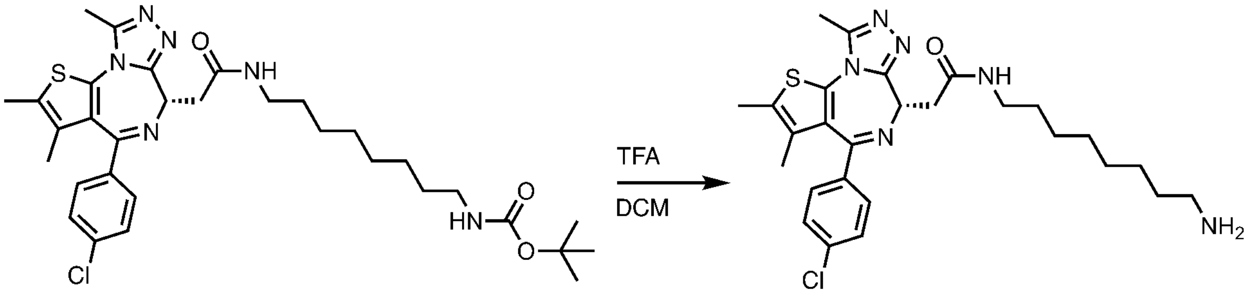

6 **7**

Compound 6 (23 mg, 0.04 mmol) was dissolved in 2 mL DCM and 0.5 mL TFA, and stirred at rt for 2 h. The reaction mixture was concentrated *in vacuo* and used without further purification (quant.). MS (ESI) m/z 527.3 $(M + H)^+$.

(*R*)-1-(2-(2-((8-(2-((*S*)-4-(4-Chlorophenyl)-2,3,9-trimethyl-6*H*-thieno[3,2-*f*][1,2,4]triazolo[4,3-*a*][1,4]diazepin-6-yl)acetamido)octyl)amino)-2-oxoethoxy)phenyl)-3-(3,4-dimethoxyphenyl)propyl (*S*)-1-((*S*)-2-(3,4,5-trimethoxyphenyl)butanoyl)piperidine-2-carboxylate (JWJ-01-255)

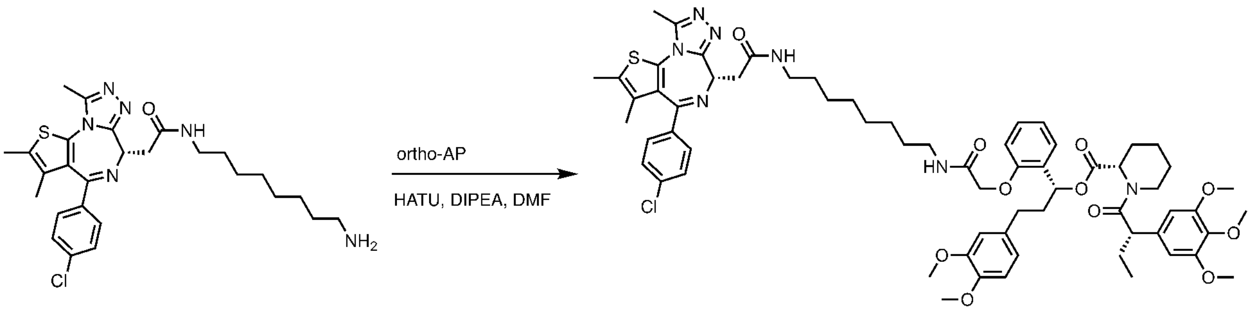

7 JWJ-01-255

Compound 7 (24 mg, 0.04 mmol), ortho-AP (23 mg, 0.04 mmol), HATU (17 mg, 0.05 mmol), DIPEA (30 µL, 0.19 mmol) were dissolved in DMF (2 mL) and stirred at rt for 16 h. The reaction mixture was filtered and purified by HPLC to afford the title compound as a while solid (3.1 mg, 6.3%) with 99% purity. ^{1}H NMR (600 MHz, DMSO-d_6) δ 8.15 (t, *J* = 5.6 Hz, 1H), 7.52–7.45 (m, 2H), 7.42 (d, *J* = 8.2 Hz, 2H), 6.89–6.72 (m, 4H), 6.66–6.58 (m, 1H), 6.55 (s, 1H), 6.03 (dd, *J* = 8.3, 4.9 Hz, 1H), 5.32 (dd, *J* = 6.0, 2.5 Hz, 1H), 4.62–4.39 (m, 4H), 3.73 (d, *J* = 4.2 Hz, 2H), 3.71 (s, 3H), 3.69 (s, 3H), 3.55 (d, *J* = 9.3 Hz, 8H), 3.28–3.15 (m, 2H), 3.15–3.01 (m, 5H), 2.59 (s, 5H), 2.40 (d, *J* = 3.6 Hz, 4H), 2.22–2.09 (m, 1H), 2.06–1.83 (m, 2H), 1.65–1.53 (m, 7H), 1.37 (ddt, *J* = 39.8, 13.6, 6.8 Hz, 6H), 1.28–1.13 (m, 12H), 0.76 (dt, *J* = 43.4, 7.3 Hz, 3H). ^{13}C NMR (125 MHz, DMSO-d_6) δ 172.51, 171.09, 169.77, 167.75, 163.73, 154.54, 153.38, 153.22, 149.13, 147.54, 137.08, 136.89, 136.71, 136.50, 136.20, 136.11, 135.92, 133.79, 132.70, 131.54, 130.74, 130.42, 130.20, 129.25, 128.98, 128.86, 126.69, 121.68, 120.48, 112.63, 112.39, 112.26, 105.54, 105.31, 70.19, 67.62, 60.28, 56.40, 56.00, 55.79, 54.35, 51.92, 49.19, 43.40, 39.02, 38.81, 38.05, 36.82, 31.11, 29.79, 29.55, 29.32, 28.60, 26.95, 26.86, 25.51, 21.03, 14.55, 13.19, 12.87, 12.77, 11.81. MS (ESI) *m/z* 1203.6 $(M + H)^+$.

tert-Butyl acetyl(3-(3-cyclopropyl-5-((2-fluoro-4-iodophenyl)amino)-6,8-dimethyl-2,4,7-trioxo-3,4,6,7-tetrahydropyrido[4,3-_d_]pyrimidin-1(2_H_)-yl)phenyl)carbamate (8)

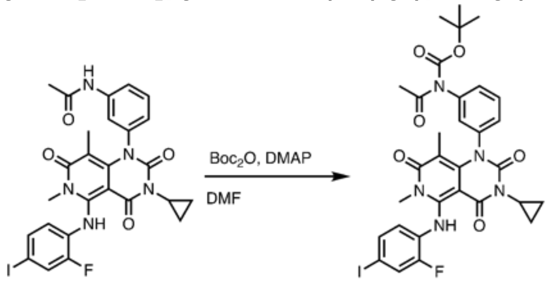

Trametinib 8

According to a reported procedure,[1] to a solution of trametinib (125 mg, 0.20 mmol), 4-(dimethylamino)pyridine (DMAP; 49.6 mg, 0.41 mmol) and DMF (1.5 mL) in an 8 mL vial, was added a solution of di-*tert*-butyl dicarbonate (Boc_2O; 133 mg, 0.61 mmol) and DMF (1.5 mL) dropwise over 1 min. The vial was sealed under nitrogen and the solution was stirred for 1 h. The solution was transferred to a 50 mL flask and concentrated to dryness to afford the title compound 8 without further purification, MS (ESI) m/z 716.2 $(M + H)^+$.

tert-Butyl (3-(3-cyclopropyl-5-((2-fluoro-4-iodophenyl)amino)-6,8-dimethyl-2,4,7-trioxo-3,4,6,7-tetrahydropyrido[4,3-_d_]pyrimidin-1(2_H_)-yl)phenyl)carbamate (9)

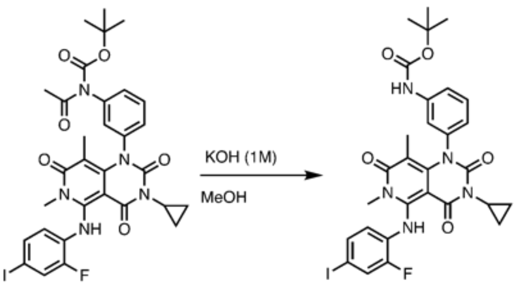

The crude solid 8 was dissolved in MeOH (1.5 mL) and then an aqueous solution of KOH (1.0 mL, 1.0 M solution, 1.0 mmol) was added. The solution was stirred for 4 h, then diluted with brine (30 mL) and extracted with DCM (3 x 15 mL). The organic extracts were pooled, dried over $MgSO_4$, filtered, and concentrated to dryness, which yielded the title compound 9 without further purification, MS (ESI) m/z 674.3 $(M + H)^+$.

1-(3-Aminophenyl)-3-cyclopropyl-5-((2-fluoro-4-iodophenyl)amino)-6,8-dimethylpyrido[4,3-*d*]pyrimidine-2,4,7(1*H*,3*H*,6*H*)-trione (10)

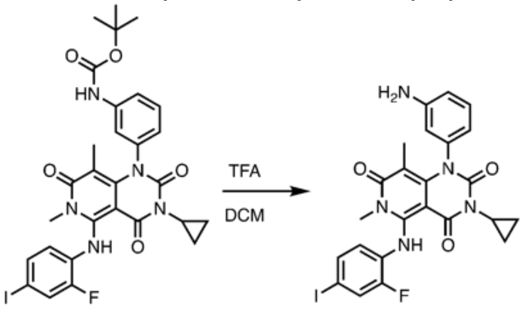

The resulting solid 9 was dissolved in TFA (3.0 mL, 39 mmol). The solution was stirred for 30 min and then concentrated to dryness from toluene (3 x 3 mL). The remaining material was partitioned between DCM (25 mL) and saturated $NaHCO_3$ solution (25 mL), and transferred to a separatory funnel. The layers were separated, and the aqueous phase was further extracted with DCM (25 mL). The organic extracts were dried over $MgSO_4$, filtered, and concentrated to dryness. The residue was purified by silica gel chromatography to yield 95 mg (81% over 3 steps) of the title compound 10 as an off-white solid. ^{1}H NMR (500 MHz, DMSO-d_6) δ 11.07 (s, 1H), 7.78 (dd, J = 10.3, 1.9 Hz, 1H), 7.56–7.52 (m, 1H), 7.05 (t, J = 7.9 Hz, 1H), 6.90 (t, J = 8.6 Hz, 1H), 6.59–6.52 (m, 2H), 6.49–6.44 (m, 1H), 5.26 (s, 2H), 3.07 (s, 3H), 2.61 (tt, J = 7.2, 4.0 Hz, 1H), 1.35 (s, 3H), 1.00–0.85 (m, 2H), 0.70–0.59 (m, 2H). MS (ESI) m/z 574.1 $(M + H)^+$.

***tert*-Butyl (2-(2-(3-((3-(3-cyclopropyl-5-((2-fluoro-4-iodophenyl)amino)-6,8-dimethyl-2,4,7-trioxo-3,4,6,7-tetrahydropyrido[4,3-*d*]pyrimidin-1(2*H*)-yl)phenyl)amino)-3-oxopropoxy)ethoxy)ethyl)carbamate (11)**

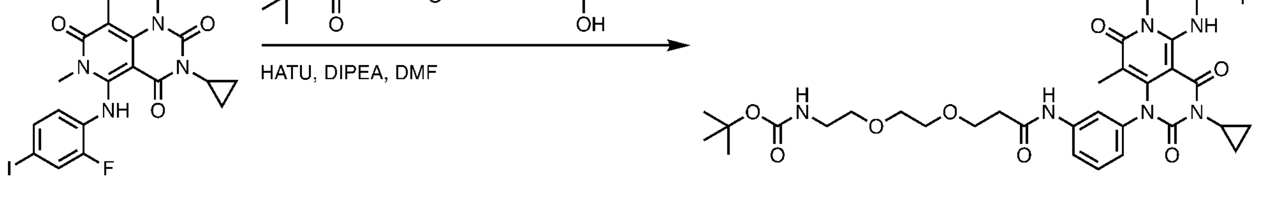

The resulting solid 10 (95 mg, 0.17 mmol), HATU (76 mg, 0.20 mmol), DIPEA (50 µL, 0.25 mmol), and 2,2-dimethyl-4-oxo-3,8,11-trioxa-5-azatetradecan-14-oic acid (51 mg, 0.18 mmol) were charged into a 8 mL vial. After adding 2 mL DMF, the solution was stirred for 2 hours. Upon the completion of the coupling reaction indicated by LCMS, the material was partitioned between DCM (25 mL) and brine (25 mL), and transferred to a separatory funnel. The layers were separated, and the aqueous phase was further extracted with DCM (25 mL). The organic extracts were dried over $MgSO_4$, filtered, and concentrated to dryness. The residue was purified by silica gel chromatography to yield 112 mg (81%) of the title compound 11. ^{1}H NMR (500 MHz, Methanol-*d*$_4$) δ 7.71 (t, *J* = 2.1 Hz, 1H), 7.67–7.63 (m, 1H), 7.58–7.50 (m, 2H), 7.38 (t, *J* = 8.1 Hz, 1H), 7.12–7.06 (m, 1H), 6.84 (t, *J* = 8.5 Hz, 1H), 6.53 (s, 1H), 3.80 (t, *J* = 6.1 Hz, 2H), 3.71 (p, *J* = 6.6 Hz, 2H), 3.62–3.60 (m, 2H), 3.60–3.57 (m, 2H), 3.47 (t, *J* = 5.6 Hz, 2H), 3.21 (q, *J* = 7.4 Hz, 2H), 3.18 (s, 3H), 3.16 (t, *J* = 5.5 Hz, 2H), 2.69 (tt, *J* = 7.3, 3.9 Hz, 1H), 2.61 (t, *J* = 6.0 Hz, 2H), 1.40 (s, 10H), 1.03 (dd, *J* = 7.0, 1.5 Hz, 2H), 0.76–0.70 (m, 2H). MS (ESI) m/z 833.4 $(M + H)^+$.

3-(2-(2-Aminoethoxy)ethoxy)-*N*-(3-(3-cyclopropyl-5-((2-fluoro-4-iodophenyl)amino)-6,8-dimethyl-2,4,7-trioxo-3,4,6,7-tetrahydropyrido[4,3-*d*]pyrimidin-1(2*H*)-yl)phenyl)propanamide (12)

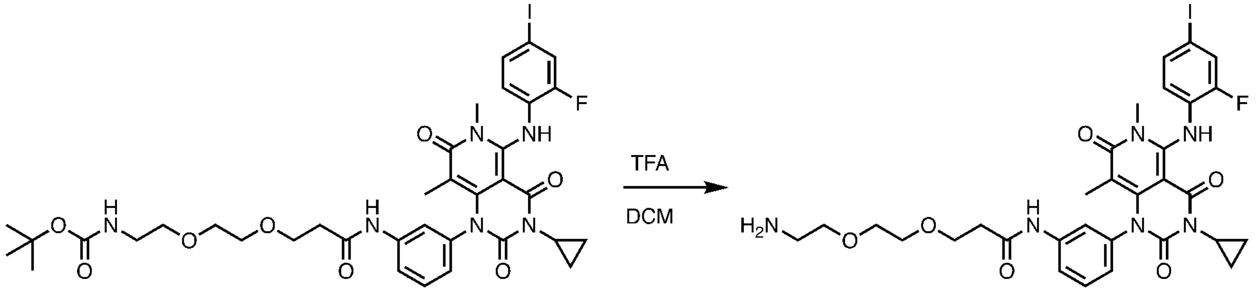

11 **12**

Compound 11 (60 mg, 0.07 mmol) was dissolved in 2 mL DCM and 1 mL TFA, and stirred at rt for 2 h. The reaction mixture was concentrated *in vacuo* and used without further purification (quant.). MS (ESI) m/z 733.2 $(M + H)^+$.

(*R*)-1-(2-(2-((2-(2-(3-((3-(3-Cyclopropyl-5-((2-fluoro-4-iodophenyl)amino)-6,8-dimethyl-2,4,7-trioxo-3,4,6,7-tetrahydropyrido[4,3-*d*]pyrimidin-1(2*H*)-yl)phenyl)amino)-3-oxopropoxy)ethoxy)ethyl)amino)-2-oxoethoxy)phenyl)-3-(3,4-dimethoxyphenyl)propyl (*S*)-1-((*S*)-2-(3,4,5-trimethoxyphenyl)butanoyl)piperidine-2-carboxylate (JWJ-01-280)

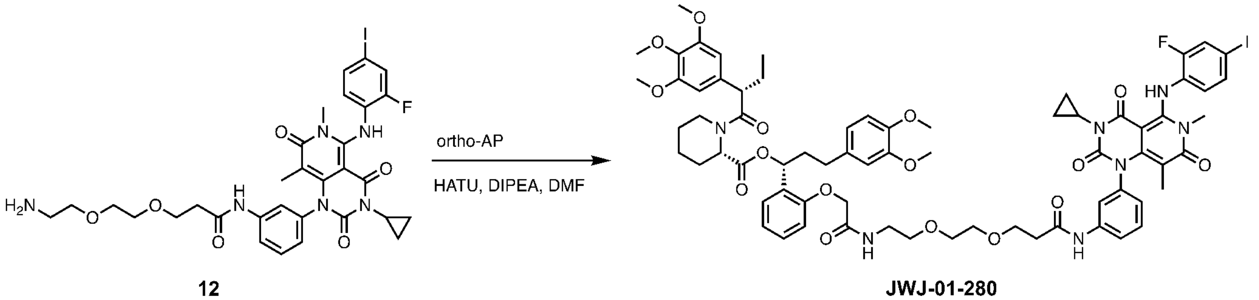

12 **JWJ-01-280**

Compound 12 (42 mg, 0.06 mmol), ortho-AP (20 mg, 0.03 mmol), HATU (13 mg, 0.04 mmol), DIPEA (25 µL, 0.14 mmol) were dissolved in DMF (2 mL) and stirred at rt for 16 h. The reaction mixture was filtered and purified by HPLC to afford the title compound as a while solid (13 mg, 29%) with 98% purity. ^{1}H NMR (500 MHz, DMSO-*d*$_6$) δ 11.07 (s, 1H), 10.11 (s, 1H), 7.84 (t, *J* = 5.7 Hz, 1H), 7.78 (dd, *J* = 10.2, 1.9 Hz, 1H), 7.64 (t, *J* = 2.1 Hz, 1H), 7.61–7.51 (m, 2H), 7.39–7.32 (m, 1H), 7.19 (ddd, *J* = 8.6, 6.7, 2.4 Hz, 1H), 7.02 (dd, *J* = 8.2, 6.0 Hz, 1H), 6.94–6.82 (m, 2H), 6.82–6.76 (m, 3H), 6.73 (d, *J* = 2.0 Hz, 1H), 6.66–6.60 (m, 1H), 6.55 (s, 1H), 6.02 (dd, *J* =

8.3, 4.8 Hz, 1H), 5.34–5.30 (m, 1H), 4.54–4.46 (m, 2H), 3.86 (t, *J* = 7.2 Hz, 2H), 3.74 (s, 2H), 3.70 (s, 3H), 3.68 (s, 3H), 3.66 (t, *J* = 6.4 Hz, 2H), 3.63 (s, 1H), 3.55 (s, 4H), 3.53 (s, 2H), 3.50–3.42 (m, 4H), 3.39 (t, *J* = 5.9 Hz, 2H), 3.30–3.20 (m, 2H), 3.06 (s, 3H), 2.65–2.57 (m, 2H), 2.57–2.52 (m, 3H), 2.44–2.35 (m, 1H), 2.15 (d, *J* = 13.0 Hz, 1H), 2.08–1.83 (m, 3H), 1.66–1.47 (m, 4H), 1.42–1.06 (m, 6H), 0.94 (q, *J* = 6.8 Hz, 2H), 0.76 (dt, *J* = 37.7, 7.3 Hz, 3H), 0.66 (dd, *J* = 7.0, 4.0 Hz, 2H). ^{13}C NMR (100 MHz, DMSO-d_6) δ 172.53, 170.96, 170.00, 168.11, 164.71, 163.36, 154.58, 153.40, 153.36, 153.19, 151.53, 151.35, 149.09, 147.49, 145.41, 140.83, 139.95, 136.45, 136.11, 134.00, 133.83, 129.34, 129.28, 129.17, 128.97, 128.73, 128.61, 126.51, 125.49, 125.28, 124.62, 121.66, 120.62, 120.46, 118.82, 112.60, 112.25, 105.52, 105.28, 102.47, 90.81, 88.76, 70.35, 70.06, 69.34, 67.61, 67.03, 60.53, 60.29, 56.41, 55.99, 55.96, 55.78, 51.94, 49.21, 43.43, 38.78, 37.59, 34.49, 31.07, 28.61, 26.81, 25.38, 21.02, 13.56, 12.81, 8.70. MS (ESI) *m/z* 1043.3 $(M + H)^+$ one fragment. HRMS (m/z): $[M+H]^+$ calcd. for $C_{69}H_{80}FIN_7O_{16}$, 1408.4685; found, 1408.4679.

Proposed fragmentation pattern

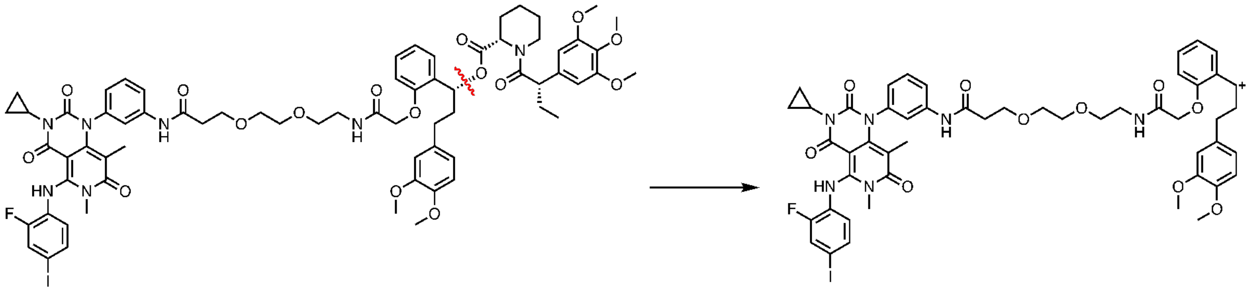

Exact Mass: 1407.46 Exact Mass: 1043.28

tert-Butyl (2-(2-(2-(3-((3-(3-cyclopropyl-5-((2-fluoro-4-iodophenyl)amino)-6,8-dimethyl-2,4,7-trioxo-3,4,6,7-tetrahydropyrido[4,3-*d*]pyrimidin-1(2*H*)-yl)phenyl)amino)-3-oxopropoxy)ethoxy)ethoxy)ethyl)carbamate (13)

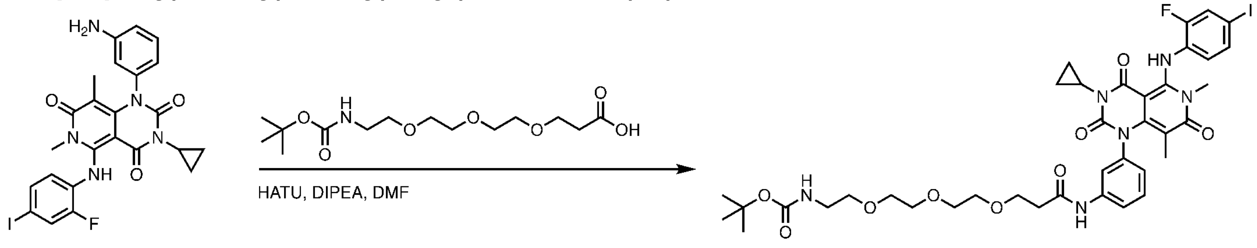

10 **13**

The resulting solid 10 (50 mg, 0.87 mmol), HATU (50 mg, 1.3 mmol), DIPEA (30 μL, 0.17 mmol), and *t*-Boc-*N*-amido-PEG$_3$-acid (31 mg, 0.096 mmol) were charged into an 8 mL vial. After adding 2 mL DMF, the solution was stirred for 2 hours. Upon the completion of the coupling reaction indicated by LCMS, the material was partitioned between DCM (25 mL) and brine (25 mL), and transferred to a separatory funnel. The layers were separated, and the aqueous phase was further extracted with DCM (25 mL). The organic extracts were dried over $MgSO_4$, filtered, and concentrated to dryness. The residue was purified by silica gel chromatography to yield 73 mg (95%) of the title compound 13.

3-(2-(2-(2-Aminoethoxy)ethoxy)ethoxy)-*N*-(3-(3-cyclopropyl-5-((2-fluoro-4-iodophenyl)amino)-6,8-dimethyl-2,4,7-trioxo-3,4,6,7-tetrahydropyrido[4,3-*d*]pyrimidin-1(2*H*)-yl)phenyl)propenamide (14)

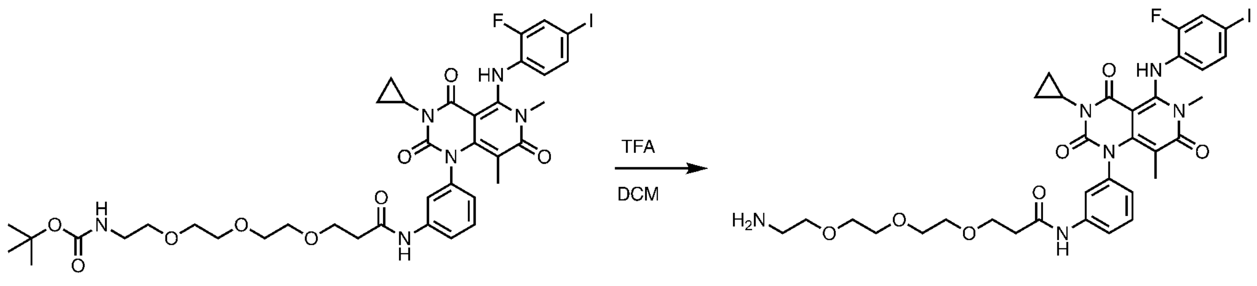

13 **14**

Compound 13 (38 mg, 0.043 mmol) was dissolved in 2 mL DCM and 1 mL TFA, and stirred at rt for 2 h. The reaction mixture was concentrated *in vacuo* and used without further purification (quant.). MS (ESI) m/z 777.2 $(M + H)^+$.

(*R*)-1-(2-((15-((3-(3-Cyclopropyl-5-((2-fluoro-4-iodophenyl)amino)-6,8-dimethyl-2,4,7-trioxo-3,4,6,7-tetrahydropyrido[4,3-*d*]pyrimidin-1(2*H*)-yl)phenyl)amino)-2,15-dioxo-6,9,12-trioxa-3-azapentadecyl)oxy)phenyl)-3-(3,4-dimethoxyphenyl)propyl (*S*)-1-((*S*)-2-(3,4,5-trimethoxyphenyl)butanoyl)piperidine-2-carboxylate (JWJ-01-295)

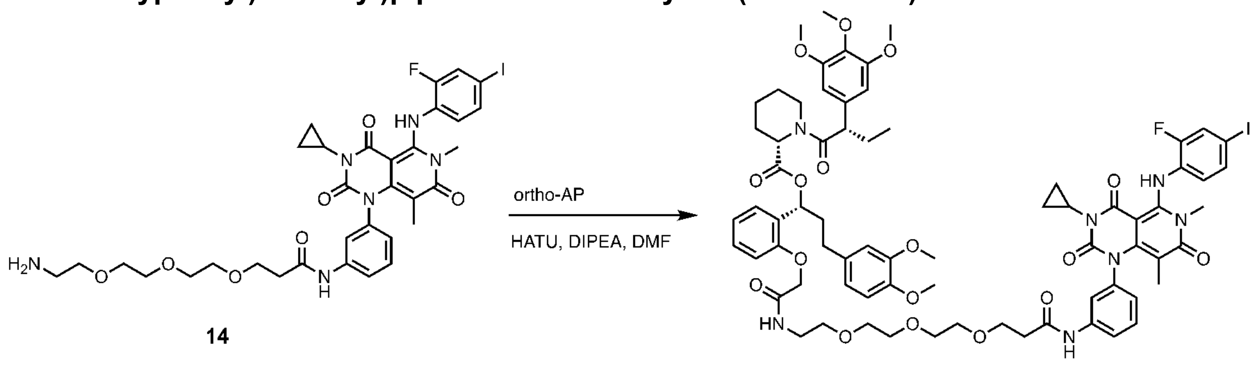

14 **JWJ-01-295**

Title compound was generated via general procedure of HATU coupling using resulting solid (38 mg, 0.043 mmol) and ortho-AP acid (23 mg, 0.033 mmol), which yielded a while solid (11.3 mg, 16%) with 98% purity. ^{1}H NMR (500 MHz, DMSO-d_6) δ 11.08 (s, 1H), 10.11 (s, 1H), 7.89 (dt, *J* = 53.1, 5.8 Hz, 1H), 7.78 (dd, *J* = 10.3, 1.9 Hz, 1H), 7.64 (t, *J* = 2.1 Hz, 1H), 7.60 (dd, *J* = 8.8, 2.0 Hz, 1H), 7.54 (dd, *J* = 8.3, 2.1 Hz, 1H), 7.35 (t, *J* = 8.0 Hz, 1H), 7.29–7.17 (m, 1H), 7.05–7.00 (m, 1H), 6.95–6.89 (m, 1H), 6.86 (d, *J* = 8.3 Hz, 1H), 6.83–6.76 (m, 3H), 6.74 (d, *J* = 2.0 Hz, 1H), 6.65–6.60 (m, 1H), 6.55 (s, 2H), 6.02 (dd, *J* = 8.3, 4.7 Hz, 1H), 5.34–5.30 (m, 1H), 4.50 (d, *J* = 3.3 Hz, 2H), 3.88–3.84 (m, 1H), 3.74 (s, 1H), 3.70 (d, *J* = 9.3 Hz, 6H), 3.68–3.65 (m, 2H), 3.63 (s, 1H), 3.54 (d, *J* = 8.3 Hz, 7H), 3.45 (dtd, *J* = 19.4, 4.8, 2.6 Hz, 9H), 3.39 (t, *J* = 5.9 Hz, 2H), 3.26 (tt, *J* = 12.2, 6.1 Hz, 2H), 3.07 (s, 3H), 2.61 (dq, *J* = 11.2, 4.0 Hz, 2H), 2.54 (t, *J* = 6.2 Hz, 3H), 2.43–2.32 (m, 1H), 2.15 (d, *J* = 13.0 Hz, 1H), 2.04–1.81 (m, 3H), 1.58 (ddd, *J* = 18.6, 14.5, 8.5 Hz, 4H), 1.37 (d, *J* = 12.4 Hz, 1H), 1.25 (s, 3H), 1.15 (t, *J* = 12.7 Hz, 1H), 0.93 (dd, *J* = 7.6, 5.9 Hz, 2H), 0.76 (dt, *J* = 37.8, 7.3 Hz, 3H), 0.69–0.64 (m, 2H). ^{13}C NMR (100 MHz, DMSO-d_6) δ δ 172.53, 170.95, 170.01, 168.10, 164.71, 163.36, 154.59, 153.40, 153.36, 153.19, 151.52, 151.35, 149.09, 147.49, 145.41, 140.83, 139.96, 136.44, 136.10, 134.51, 133.83, 129.34, 129.27, 129.17, 128.73, 128.62, 126.50, 125.48, 124.61, 121.66, 120.60, 120.45, 112.60, 112.55, 112.25, 105.51, 102.47, 90.81, 88.83, 88.76, 70.37, 70.18, 70.15, 70.07, 69.33, 67.63, 67.03, 60.53, 60.29, 56.41, 55.98, 55.96, 55.78, 51.94, 49.21, 43.43, 38.79, 37.60, 36.91, 34.50, 31.08, 28.61, 28.25, 26.81, 25.38, 21.03, 13.56, 12.81, 8.70. MS (ESI) *m/z* 1087.5 $(M + H)^+$ one fragment.

Proposed fragmentation pattern

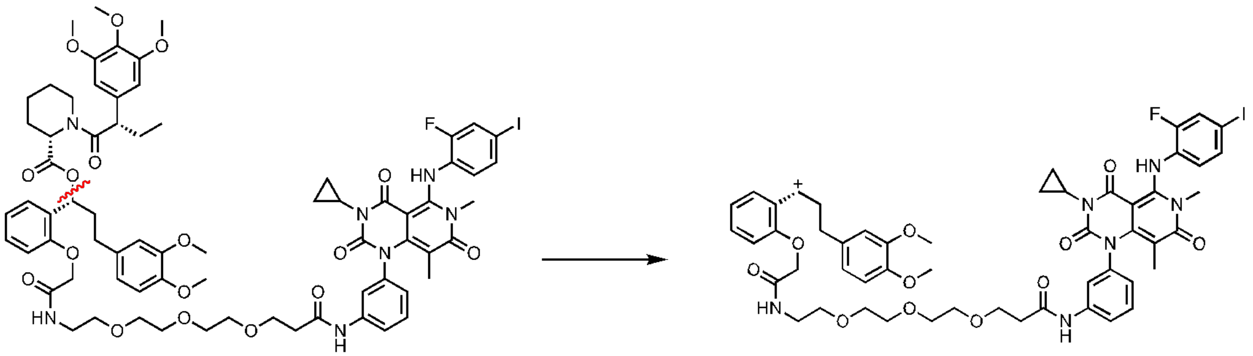

Exact Mass: 1451.49 → Exact Mass: 1087.31

tert-Butyl (9-((3-(3-cyclopropyl-5-((2-fluoro-4-iodophenyl)amino)-6,8-dimethyl-2,4,7-trioxo-3,4,6,7-tetrahydropyrido[4,3-d]pyrimidin-1(2H)-yl)phenyl)amino)-9-oxononyl)carbamate (15)

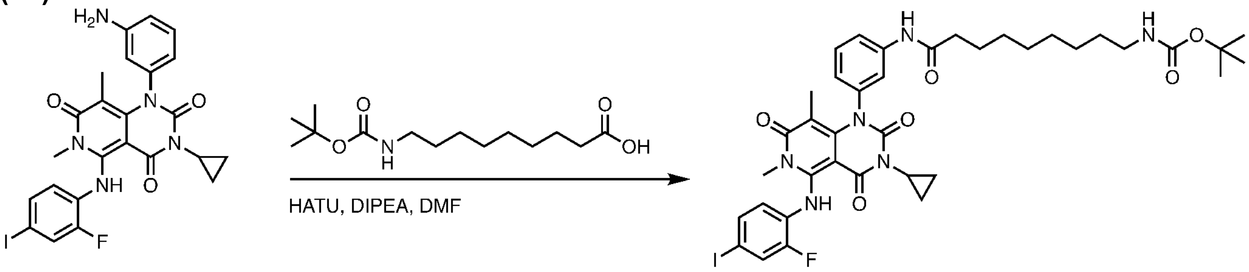

10 **15**

The resulting solid 10 (20 mg, 0.035mmol), HATU (20 mg, 0.020 mmol), DIPEA (18 µL, 0.11 mmol), and 9-((*tert*-butoxycarbonyl)amino)nonanoic acid (11 mg, 0.038 mmol) were charged into an 8 mL vial. After adding 2 mL DMF, the solution was stirred for 2 hours. Upon the completion of the coupling reaction indicated by LCMS, the material was partitioned between DCM (25 mL) and brine (25 mL), and transferred to a separatory funnel. The layers were separated, and the aqueous phase was further extracted with DCM (25 mL). The organic extracts were dried over $MgSO_4$, filtered, and concentrated to dryness. The residue was purified by silica gel chromatography to yield 25 mg (86%) of the title compound 15.

9-Amino-N-(3-(3-cyclopropyl-5-((2-fluoro-4-iodophenyl)amino)-6,8-dimethyl-2,4,7-trioxo-3,4,6,7-tetrahydropyrido[4,3-d]pyrimidin-1(2H)-yl)phenyl)nonanamide (16)

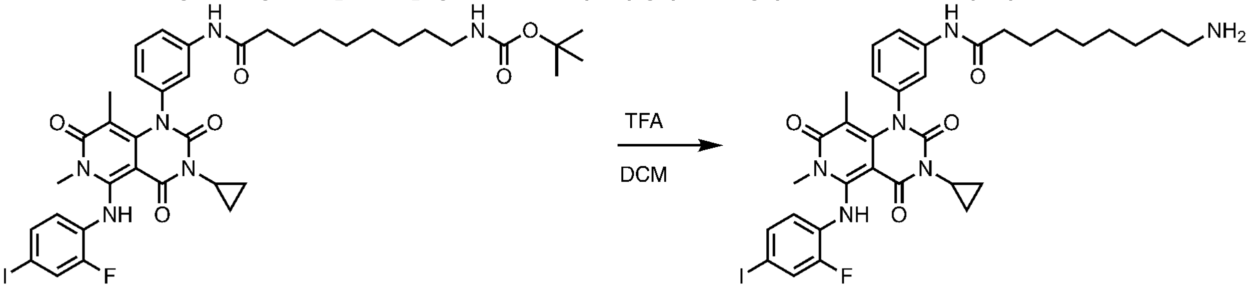

15 **16**

Compound 15 (25 mg, 0.030 mmol) was dissolved in 2 mL DCM and 1 mL TFA, and stirred at rt for 2 h. The reaction mixture was concentrated *in vacuo* and used without further purification (quant.). MS (ESI) m/z 777.2 $(M + H)^+$.

(R)-1-(2-(2-((9-((3-(3-Cyclopropyl-5-((2-fluoro-4-iodophenyl)amino)-6,8-dimethyl-2,4,7-trioxo-3,4,6,7-tetrahydropyrido[4,3-d]pyrimidin-1(2H)-yl)phenyl)amino)-9-oxononyl)amino)-2-oxoethoxy)phenyl)-3-(3,4-dimethoxyphenyl)propyl (S)-1-((S)-2-(3,4,5-trimethoxyphenyl)butanoyl)piperidine-2-carboxylate (JWJ-01-340)

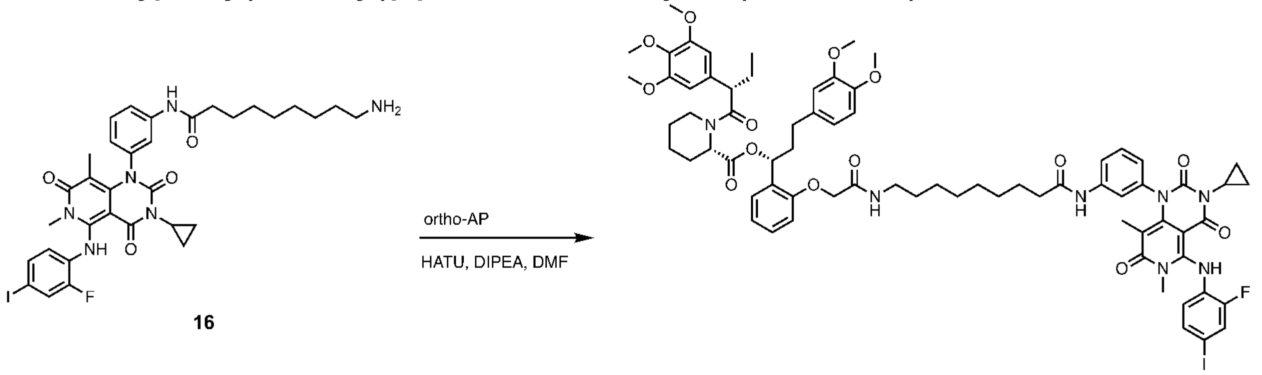

16 **JWJ-01-340**

Title compound was generated via general procedure of HATU coupling using resulting solid (22 mg, 0.030 mmol) and ortho-AP acid (16 mg, 0.023 mmol), which yielded a while solid (10.6 mg, 23%) with 99% purity. ^{1}H NMR (500 MHz, DMSO-d_6) δ 11.08 (s, 1H), 10.03 (s, 1H), 7.78 (dd, J = 10.3, 2.0 Hz, 1H), 7.67 (t, J = 5.8 Hz, 1H), 7.63 (t, J = 2.1 Hz, 1H), 7.60 (dd, J = 8.1, 2.2 Hz, 1H), 7.54 (dd, J = 8.4, 1.9 Hz, 1H), 7.37–7.32 (m, 1H), 7.20 (ddd, J = 8.7, 6.7, 2.4 Hz, 1H), 7.04–7.00 (m, 1H), 6.96–6.86 (m, 2H), 6.85–6.77 (m, 3H), 6.66–6.60 (m, 1H), 6.55 (s, 1H), 6.02 (dd, J = 8.3, 4.9 Hz, 1H), 5.32 (dd, J = 6.0, 2.4 Hz, 1H), 4.49 (q, J = 14.7 Hz, 2H), 4.05 (d, J = 13.3 Hz, 2H), 3.86 (t, J = 7.2 Hz, 4H), 3.73 (s, 2H), 3.69 (d, J = 10.0 Hz, 6H), 3.63 (s, 1H), 3.54 (d, J = 7.3 Hz, 7H), 3.07 (s, 4H), 2.64–2.55 (m, 2H), 2.44–2.33 (m, 1H), 2.28 (t, J = 7.4 Hz, 2H), 2.15 (d, J = 14.0 Hz, 1H), 2.05–1.84 (m, 3H), 1.58 (ddd, J = 22.5, 16.2, 10.2 Hz, 5H), 1.42–1.29 (m, 3H), 1.28–1.11 (m, 12H), 0.97–0.91 (m, 2H), 0.76 (dt, J = 36.7, 7.3 Hz, 3H), 0.66 (p, J = 5.7, 5.1 Hz, 2H). ^{13}C NMR (100 MHz, DMSO-d_6) δ 172.49, 172.05, 171.08, 167.75, 164.72, 163.36, 155.89, 154.90, 154.51, 153.40, 153.36, 153.20, 151.53, 151.36, 149.10, 147.51, 145.41, 140.81, 140.13, 136.44, 136.09, 134.55, 133.75, 129.23, 128.73, 128.61, 126.68, 125.48, 125.27, 124.51, 121.68, 120.56, 120.46, 118.83, 112.58, 112.37, 112.21, 105.49, 102.48, 90.80, 88.81, 88.74, 70.16, 67.59, 60.52, 60.27, 56.39, 55.98, 55.94, 55.77, 51.90, 49.19, 43.41, 40.90, 38.79, 36.88, 34.49, 31.09, 29.52, 29.33, 29.20, 28.60, 26.83, 25.52, 25.37, 21.03, 13.54, 12.78, 8.70. MS (ESI) m/z 1039.5 $(M + H)^+$ one fragment.

Proposed fragmentation pattern

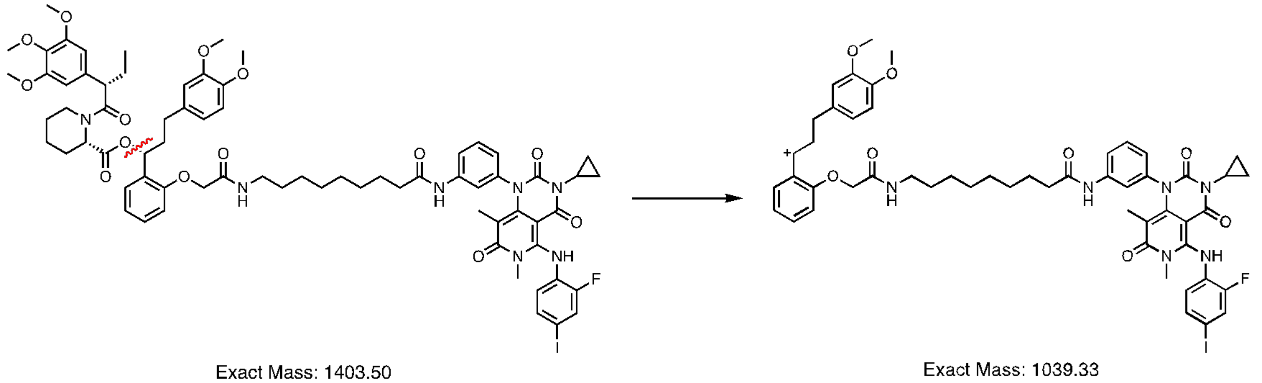

(2-(2-(2-(2-Azidoethoxy)ethoxy)ethoxy)ethyl)sulfamic acid (18)

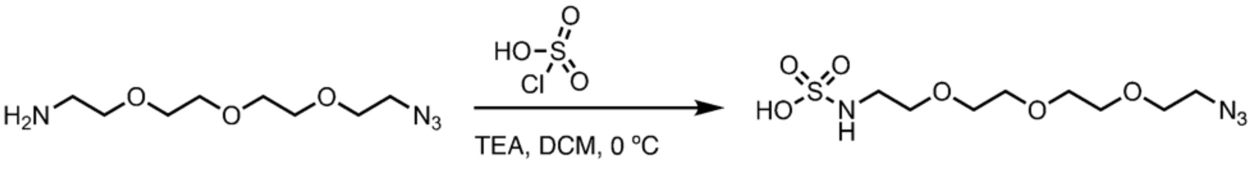

17 18

According to a reported procedure,[2] to a solution of azido-PEG$_3$-amine 17 (100 mg, 0.46 mmol) and TEA (0.58 mL, 4.2 mmol) in anhydrous DCM (2 mL) at 0 °C was added slowly chlorosulfuric acid (0.34 mL, 0.50 mmol). The resulting mixture was concentrated under reduced pressure, which yielded the desired sulfamic acid 18 as a TEA salt quantitatively. The acid was used directly in the next step without further purification. MS (ESI) m/z 297.2 $(M - H)^-$.

(2-(2-(2-(2-Azidoethoxy)ethoxy)ethoxy)ethyl)sulfamoyl chloride (19)

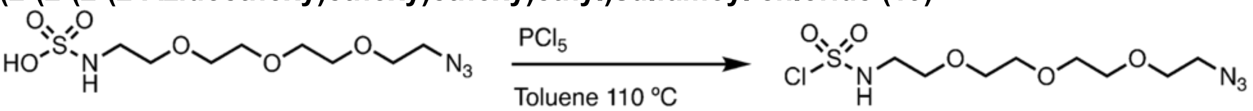

18 19

To a suspension of sulfamic acid (45 mg, 0.11 mmol) in anhydrous toluene (2 mL) at room temperature was added PCl_5 (26 mg, 0.12 mmol). The resulting mixture was allowed to heat at 110 °C for 3 hours. After cooling to room temperature, all the solid was removed via filtration. The filtrate was then concentrated under reduced vacuum, which yielded the desired chloride intermediate 19 for the next step.

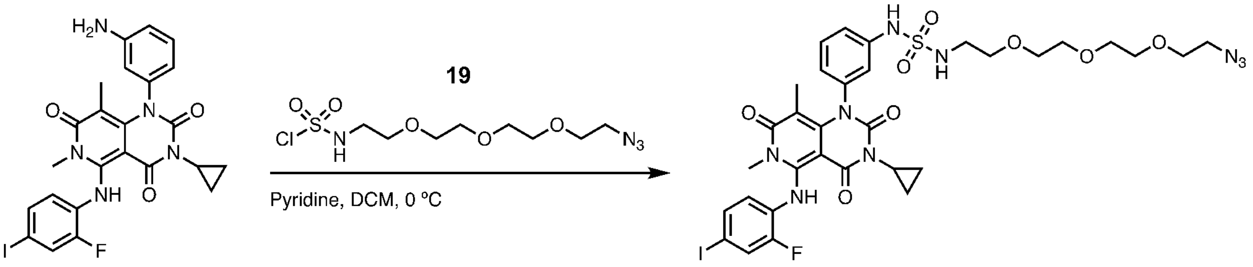

10 20

To a solution of intermediate 10 (48 mg, 0.084 mmol) in DCM (2 mL), pyridine (0.027 mL, 0.34 mmol) was added. The resulting mixture was cooled to 0 °C before the addition of chloride 19 (45 mg, 0.11 mmol) solution in DCM (2 mL) dropwise. The mixture was further stirred for 0.5 hour, which gave a clear yellow solution. Upon the completion of the coupling reaction indicated by LCMS, the crude material was purified via prep HPLC to yield the desired product 20 (25 mg, 30%). ^{1}H NMR (500 MHz, DMSO-d_6) δ 11.09 (s, 1H), 9.85 (s, 1H), 7.79 (dd, *J* = 10.0, 1.8 Hz, 1H), 7.63 (t, *J* = 5.9 Hz, 1H), 7.57–7.52 (m, 1H), 7.35 (t, *J* = 8.1 Hz, 1H), 7.20–7.16 (m, 1H), 7.11 (t, *J* = 2.1 Hz, 1H), 7.01 (d, *J* = 8.2 Hz, 1H), 6.92 (t, *J* = 8.6 Hz, 1H), 3.61–3.56 (m, 3H), 3.56–3.52 (m, 3H), 3.50 (dd, *J* = 5.6, 3.1 Hz, 2H), 3.47 (dd, *J* = 6.0, 3.7 Hz, 2H), 3.45–3.41 (m, 2H), 3.38 (s, 2H), 3.07 (s, 3H), 2.95 (q, *J* = 6.2 Hz, 2H), 2.62 (q, *J* = 3.4 Hz, 1H), 1.25 (s, 3H), 0.95 (d, *J* = 7.2 Hz, 2H), 0.66 (s, 2H). MS (ESI) m/z 854.4 $(M + H)^+$.

(*R*)-3-(3,4-Dimethoxyphenyl)-1-(2-(2-oxo-2-(prop-2-yn-1-ylamino)ethoxy)phenyl)propyl (*S*)-1-((*S*)-2-(3,4,5-trimethoxyphenyl)butanoyl)piperidine-2-carboxylate (21)

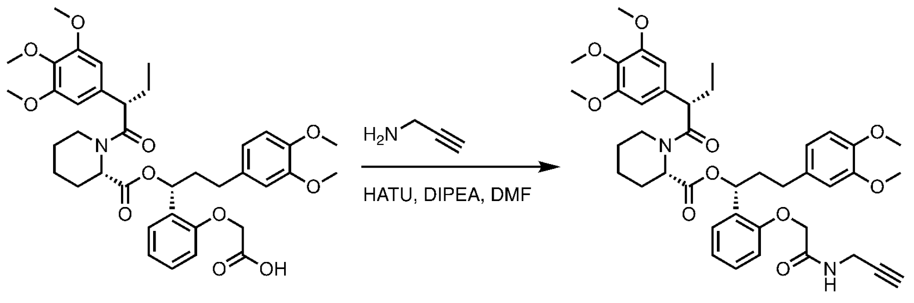

ortho-AP **21**

The ortho-AP (30 mg, 0.044 mmol), HATU (26 mg, 0.064 mmol), DIPEA (26 µL, 0.13 mmol), and propargylamine (3 µL, 0.048 mmol) were charged into an 8 mL vial. After adding 2 mL DMF, the solution was stirred overnight. Upon the completion of the coupling reaction indicated by LCMS, the residue was purified by prep HPLC to yield the desired product (3.2 mg, 11%). MS (ESI) m/z 731.4 $(M + H)^+$.

(*R*)-1-(2-(2-(((1-(2-(2-(2-(2-((*N*-(3-(3-Cyclopropyl-5-((2-fluoro-4-iodophenyl)amino)-6,8-dimethyl-2,4,7-trioxo-3,4,6,7-tetrahydropyrido[4,3-*d*]pyrimidin-1(2*H*)-yl)phenyl)sulfamoyl)amino)ethoxy)ethoxy)ethoxy)ethyl)-1*H*-1,2,3-triazol-4-yl)methyl)amino)-2-oxoethoxy)phenyl)-3-(3,4-dimethoxyphenyl)propyl (*S*)-1-((*S*)-2-(3,4,5-trimethoxyphenyl)butanoyl)piperidine-2-carboxylate (JWJ-01-348)

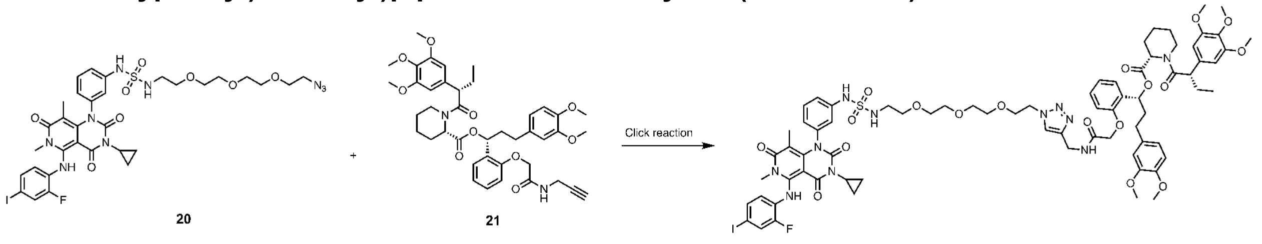

20 21 **JWJ-01-348**

Intermediate 20 (14 mg, 0.016 mmol) and 21 (12 mg, 0.016 mmol) were charged into an 8 mL vial before the addition of DMF (1 mL). Copper (II) sulfate pentahydrate (0.41 mg 1.6 µmol) and sodium ascorbate (0.65 mg 3.3 µmol) were dissolved in DI water (0.1 mL), sonicated for 15 minutes, and added to the DMF solution. Upon the completion of the click reaction indicated by LCMS, the residue was purified by prep HPLC to yield the desired product 20 (10 mg, 60%) with 99% purity. ^{1}H NMR (500 MHz, DMSO-d_6) δ 11.09 (s, 1H), 9.85 (s, 1H), 8.44 (dt, *J* = 36.8, 5.8 Hz, 1H), 7.84 (d, *J* = 10.2 Hz, 1H), 7.78 (dd, *J* = 10.3, 1.9 Hz, 1H), 7.63 (t, *J* = 5.9 Hz, 1H), 7.57–7.51 (m, 1H), 7.33 (t, *J* = 8.1 Hz, 1H), 7.28–7.16 (m, 2H), 7.12 (d, *J* = 2.2 Hz, 1H), 7.03–6.97 (m, 1H), 6.95–6.88 (m, 1H), 6.87–6.82 (m, 1H), 6.82–6.72 (m, 3H), 6.64–6.60 (m, 1H), 6.55 (s, 2H), 6.03 (dd, *J* = 8.3, 4.6 Hz, 1H), 5.31 (d, *J* = 6.4 Hz, 1H), 4.57 (d, *J* = 37.6 Hz, 2H), 4.44 (q, *J* = 5.6 Hz, 2H), 4.36 (dt, *J* = 11.0, 5.7 Hz, 2H), 4.04 (d, *J* = 13.3 Hz, 1H), 3.86 (t, *J* = 7.2 Hz, 1H), 3.77–3.73 (m, 4H), 3.68 (d, *J* = 2.5 Hz, 6H), 3.54 (s, 6H), 3.53 (s, 3H), 3.47 (dd, *J* = 5.8, 3.1 Hz, 3H), 3.44 (d, *J* = 3.3 Hz, 2H), 3.42–3.39 (m, 3H), 3.35 (t, *J* = 6.3 Hz, 2H), 3.06 (s, 2H), 2.94 (q, *J* = 6.2 Hz, 2H), 2.60 (dq, *J* = 10.8, 3.4 Hz, 2H), 2.46 (d, *J* = 4.8 Hz, 1H), 2.43–2.33 (m, 1H), 2.13 (d, *J* = 13.7 Hz, 1H), 2.06–1.81 (m, 3H), 1.66–1.48 (m, 4H), 1.36 (d, *J* = 11.4 Hz, 1H), 1.24 (s, 3H), 1.11 (d, *J* = 13.2 Hz, 1H), 0.94 (d, *J* = 7.1 Hz, 2H), 0.76 (dt, *J* = 38.2, 7.3 Hz, 3H), 0.68–0.63 (m, 2H). ^{13}C NMR (100 MHz, DMSO-d_6) δ 172.53, 170.93, 168.07, 164.72, 163.36, 154.64, 153.41, 153.36, 153.19, 151.43, 151.39, 149.08, 147.47, 145.28, 141.10, 139.79, 136.44, 136.11, 134.52, 129.48, 129.44, 129.11, 128.70, 128.59, 126.46, 125.51, 125.28, 123.92, 123.63, 121.63, 120.45, 119.26, 117.59, 112.62, 112.54, 112.26, 105.53, 105.29, 102.50, 90.68, 88.88, 88.81, 70.47, 70.12, 70.10, 70.02, 69.26, 69.22, 67.63, 60.53, 60.29, 56.41, 55.99, 55.78, 51.95, 49.79, 49.22, 43.42, 42.28,

37.00, 34.52, 31.07, 28.62, 26.80, 25.48, 25.37, 21.01, 13.43, 12.91, 12.82, 8.72. MS (ESI) m/z 793.2 $(M + 2H)^{2+}/2$.

(*R*)-1-(2-(2-(((1-(2-(2-(2-((*N*-(3-(3-Cyclopropyl-5-((2-fluoro-4-iodophenyl)amino)-6,8-dimethyl-2,4,7-trioxo-3,4,6,7-tetrahydropyrido[4,3-*d*]pyrimidin-1(2*H*)-yl)phenyl)sulfamoyl)amino)ethoxy)ethoxy)ethyl)-1*H*-1,2,3-triazol-4-yl)methyl)amino)-2-oxoethoxy)phenyl)-3-(3,4-dimethoxyphenyl)propyl (*S*)-1-((*S*)-2-(3,4,5-trimethoxyphenyl)butanoyl)piperidine-2-carboxylate (JWJ-01-354)

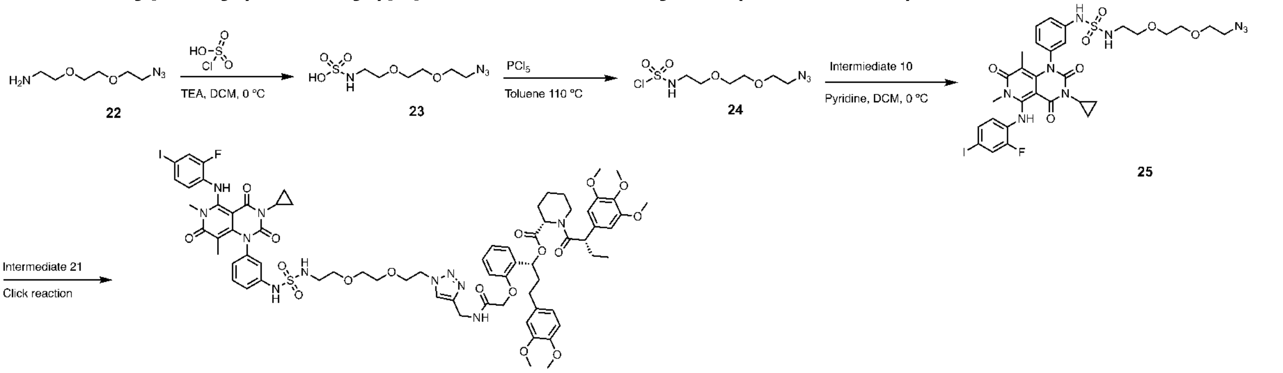

JWJ-01-354

By employment of the similar procedure to JWJ-01-348, from the corresponding azide 25 (22 mg, 0.027 mmol), the title compound was prepared (18.3 mg, 40%) with 99% purity. ^{1}H NMR (400 MHz, DMSO-d_6) δ 11.10 (s, 1H), 9.87 (s, 1H), 8.42 (t, *J* = 5.8 Hz, 1H), 7.84 (d, *J* = 7.4 Hz, 1H), 7.78 (dd, *J* = 10.3, 1.9 Hz, 1H), 7.65 (t, *J* = 5.9 Hz, 1H), 7.54 (dd, *J* = 8.5, 2.0 Hz, 1H), 7.34 (t, *J* = 8.1 Hz, 1H), 7.18 (ddt, *J* = 8.8, 4.6, 2.5 Hz, 2H), 7.14–7.10 (m, 1H), 7.00 (dd, *J* = 7.9, 1.9 Hz, 1H), 6.91 (t, *J* = 8.7 Hz, 1H), 6.86–6.82 (m, 1H), 6.82–6.75 (m, 3H), 6.73 (d, *J* = 2.0 Hz, 1H), 6.62 (td, *J* = 4.4, 3.5, 1.8 Hz, 1H), 6.55 (s, 2H), 6.04 (dd, *J* = 8.2, 4.6 Hz, 1H), 5.32 (dd, *J* = 5.8, 2.4 Hz, 1H), 4.58 (d, *J* = 30.0 Hz, 2H), 4.44 (t, *J* = 5.2 Hz, 2H), 4.37 (dt, *J* = 14.0, 4.5 Hz, 2H), 4.04 (d, *J* = 13.1 Hz, 1H), 3.87 (t, *J* = 7.1 Hz, 1H), 3.77–3.72 (m, 4H), 3.69 (d, *J* = 2.6 Hz, 7H), 3.63 (s, 1H), 3.54 (d, *J* = 5.6 Hz, 8H), 3.45 (dd, *J* = 6.1, 3.5 Hz, 2H), 3.39 (dt, *J* = 6.4, 2.8 Hz, 2H), 3.34 (t, *J* = 6.2 Hz, 2H), 3.06 (s, 3H), 2.95 (q, *J* = 6.1 Hz, 2H), 2.61 (tt, *J* = 7.1, 3.9 Hz, 2H), 2.14 (d, *J* = 13.2 Hz, 1H), 2.08–1.80 (m, 2H), 1.67–1.45 (m, 4H), 1.38 (t, *J* = 12.0 Hz, 1H), 1.24 (s, 4H), 0.97–0.90 (m, 2H), 0.77 (dt, *J* = 29.9, 7.2 Hz, 3H), 0.66 (t, *J* = 5.2 Hz, 2H). ^{13}C NMR (100 MHz, DMSO-d_6) δ 172.53, 170.93, 168.08, 164.72, 163.36, 154.64, 153.41, 153.36, 153.19, 151.44, 151.39, 149.08, 147.47, 145.28, 141.10, 139.79, 136.45, 136.11, 134.52, 133.90, 129.48, 129.44, 129.10, 128.70, 128.59, 126.46, 125.51, 123.92, 123.64, 121.64, 120.45, 119.23, 117.56, 112.62, 112.27, 105.53, 105.29, 102.49, 90.68, 88.88, 88.81, 70.47, 69.98, 69.89, 69.25, 69.19, 67.63, 60.53, 60.29, 56.41, 55.99, 55.97, 55.78, 51.95, 49.77, 49.23, 43.42, 42.28, 40.65, 36.99, 34.53, 31.07, 28.62, 25.37, 21.01, 13.43, 12.91, 12.82, 8.72. MS (ESI) m/z 770.8 $(M + 2H)^{2+}/2$. MS (ESI) *m/z* 676.0 $(M + 2H)^{2+}/2$. HRMS (m/z): $[M+H]^+$ calcd. for $C_{71}H_{84}FIN_{11}O_{17}S$, 1540.4791; found, 1540.4798.

(*R*)-1-(2-((1-(4-((9-Chloro-7-(2-fluoro-6-methoxyphenyl)-5*H*-benzo[*c*]pyrimido[4,5-*e*]azepin-2-yl)amino)-2-methoxyphenyl)-1,15-dioxo-5,8,11-trioxa-2,14-diazahexadecan-16-yl)oxy)phenyl)-3-(3,4-dimethoxyphenyl)propyl (*S*)-1-((*S*)-2-(3,4,5-trimethoxyphenyl)butanoyl)piperidine-2-carboxylate (JWJ-01-342)

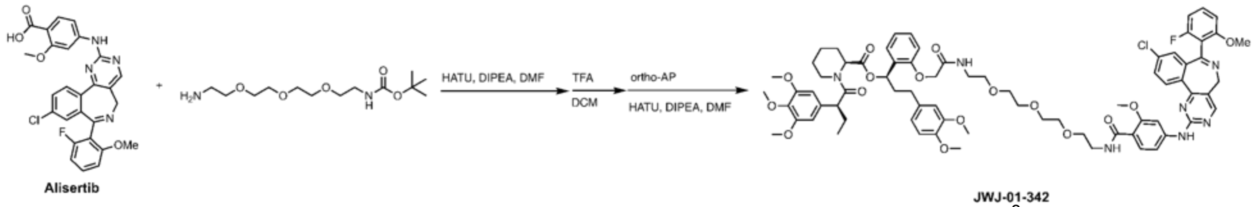

Alisertib

JWJ-01-342

Alisertib (20 mg, 0.039 mmol, prepared based on the reported procedures[3]), *t*-Boc-*N*-amido-PEG$_3$-amine (11 mg, 0.042 mmol), HATU (22 mg, 0.058 mmol), and DIPEA (0.013 mL, 77 mmol) were charged into an 8 mL scintillation vial. After the addition of DMF (2 mL), the mixture was stirred for 12 hours. Upon the consumption of the alisertib, the mixture was partitioned between EA (25 mL) and brine (25 mL). The layers were separated in a separator funnel, and the aqueous phase was further extracted with EA (25 mL). The organic extracts were dried over $MgSO_4$, filtered, and concentrated to dryness. The residue was purified by silica gel chromatography to yield the desired intermediate, which was treated with a 1:1 (v/v) mixture of DCM/TFA (5 mL/mmol) for 30 min. Upon the completion of the removal of the Boc group indicated by UPLC, excess TFA and DCM were evaporated under reduced pressure. After putting on the high vacuum for additional 2 hours to fully remove TFA, the residue was dissolved in DMF (1 mL). HATU (1.5 equiv), DIPEA (2 equiv), and the ortho-AP acid (1 equiv) were added to the solution and stirred overnight. Upon the completion of the HATU coupling indicated by UPLC, the mixture was diluted with DMSO to 5 mL and filtered through a 13 mm syringe filter (0.45 um, PTFE). The resulting filtrate was loaded and purified via the preparative HPLC, which yielded the title compound (19.8 mg, 46%) with 99% purity. ^{1}H NMR (500 MHz, DMSO-*d*$_6$) δ 10.22 (s, 1H), 8.72 (s, 1H), 8.31 (d, *J* = 8.6 Hz, 1H), 8.16 (t, *J* = 5.4 Hz, 1H), 7.96 (d, *J* = 15.7 Hz, 1H), 7.90–7.80 (m, 3H), 7.46–7.39 (m, 2H), 7.34–7.16 (m, 2H), 7.03–6.90 (m, 1H), 6.89–6.76 (m, 4H), 6.73 (d, *J* = 2.1 Hz, 1H), 6.65–6.59 (m, 1H), 6.54 (s, 2H), 6.02 (dd, *J* = 8.4, 4.7 Hz, 1H), 5.34–5.29 (m, 1H), 4.88 (s, 1H), 4.50 (d, *J* = 3.4 Hz, 2H), 4.42 (d, *J* = 12.6 Hz, 1H), 4.04 (d, *J* = 13.5 Hz, 2H), 3.92 (s, 3H), 3.86 (t, *J* = 7.3 Hz, 2H), 3.73 (s, 2H), 3.69 (d, *J* = 10.8 Hz, 6H), 3.64 (d, *J* = 12.1 Hz, 1H), 3.55 (s, 5H), 3.54–3.49 (m, 7H), 3.49–3.42 (m, 6H), 3.39 (t, *J* = 5.9 Hz, 2H), 3.26 (tt, *J* = 11.6, 5.9 Hz, 3H), 2.64–2.56 (m, 1H), 2.49–2.44 (m, 1H), 2.43–2.33 (m, 1H), 2.15 (d, *J* = 13.1 Hz, 1H), 2.07–1.83 (m, 3H), 1.58 (ddd, *J* = 19.8, 14.7, 9.4 Hz, 4H), 1.36 (d, *J* = 11.1 Hz, 1H), 1.27–0.96 (m, 2H), 0.76 (dt, *J* = 37.0, 7.4 Hz, 3H). ^{13}C NMR (100 MHz, DMSO-*D*$_6$) δ 172.52, 170.95, 170.71, 168.09, 164.80, 161.32, 159.96, 159.01, 158.88, 158.64, 158.42, 158.36, 157.77, 154.96, 154.59, 153.37, 153.19, 149.10, 147.55, 147.49, 145.17, 136.76, 136.67, 136.46, 136.10, 135.23, 134.00, 133.84, 132.14, 131.49, 129.35, 129.15, 126.51, 123.78, 121.66, 120.45, 114.85, 112.61, 112.55, 112.25, 111.02, 108.57, 105.52, 105.29, 101.95, 70.35, 70.23, 70.15, 70.09, 69.59, 69.35, 67.65, 60.51, 60.27, 56.39, 56.22, 55.97, 55.93, 55.77, 51.95, 49.65, 49.22, 43.42, 38.78, 36.92, 31.08, 28.61, 26.82, 25.48, 21.02, 12.88, 12.79. MS (ESI) *m/z* 685.0 $(M + 2H)^{2+}/2$. HRMS (m/z): $[M+H]^+$ calcd. for $C_{73}H_{83}ClFN_7O_{16}Na$, 1390.5461; found, 1390.5462.

(*R*)-1-(2-((1-(4-((9-Chloro-7-(2-fluoro-6-methoxyphenyl)-5*H*-benzo[*c*]pyrimido[4,5-e]azepin-2-yl)amino)-2-methoxyphenyl)-1,12-dioxo-5,8-dioxa-2,11-diazatridecan-13-yl)oxy)phenyl)-3-(3,4-dimethoxyphenyl)propyl (*S*)-1-((*S*)-2-(3,4,5-trimethoxyphenyl)butanoyl)piperidine-2-carboxylate (JWJ-01-343)

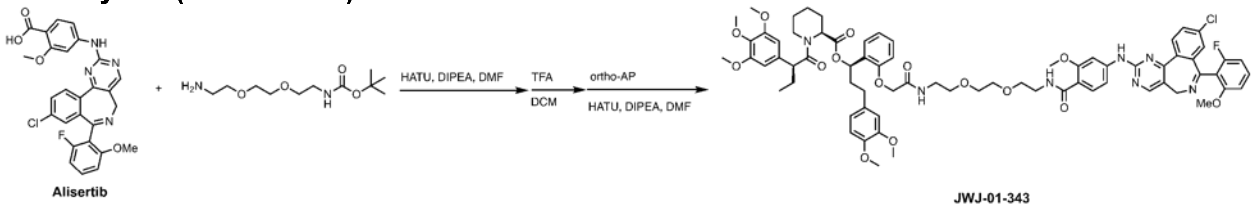

Alisertib

JWJ-01-343

By employment of the similar procedure to JWJ-01-342, from alisertib (15 mg, 0.029 mmol), the title compound was prepared (7.9 mg, 18%) with 99% purity. ^{1}H NMR (500 MHz, DMSO-*d*$_6$) δ

10.21 (s, 1H), 8.72 (s, 1H), 8.30 (d, *J* = 8.5 Hz, 1H), 8.16 (t, *J* = 5.4 Hz, 1H), 7.97 (d, *J* = 10.8 Hz, 1H), 7.89–7.80 (m, 3H), 7.41 (ddd, *J* = 8.5, 4.3, 2.4 Hz, 2H), 7.30–7.15 (m, 2H), 6.85 (d, *J* = 8.3 Hz, 1H), 6.79 (qt, *J* = 7.7, 3.8 Hz, 3H), 6.73 (d, *J* = 2.0 Hz, 1H), 6.69–6.60 (m, 2H), 6.54 (s, 2H), 6.02 (dd, *J* = 8.2, 4.8 Hz, 1H), 5.32 (dd, *J* = 6.0, 2.4 Hz, 1H), 4.93 (d, *J* = 56.1 Hz, 1H), 4.60–4.36 (m, 3H), 3.91 (s, 4H), 3.86 (d, *J* = 7.0 Hz, 2H), 3.73 (s, 2H), 3.69 (d, *J* = 12.2 Hz, 6H), 3.62 (s, 1H), 3.54 (d, *J* = 8.6 Hz, 8H), 3.51 (ddd, *J* = 6.8, 4.5, 2.1 Hz, 5H), 3.44 (dt, *J* = 15.4, 5.8 Hz, 4H), 3.28 (tp, *J* = 13.3, 6.9, 6.0 Hz, 3H), 2.59 (t, *J* = 13.0 Hz, 1H), 2.49–2.45 (m, 1H), 2.38 (dt, *J* = 16.9, 11.5 Hz, 1H), 2.15 (d, *J* = 13.3 Hz, 1H), 2.04–1.81 (m, 3H), 1.58 (dt, *J* = 21.2, 14.0 Hz, 4H), 1.41–1.29 (m, 1H), 1.26–0.96 (m, 2H), 0.76 (dt, *J* = 36.8, 7.3 Hz, 3H). MS (ESI) *m/z* 663.1 (M + 2H)$^{2+}$/2.

(*R*)-1-(2-(2-((8-(4-((9-Chloro-7-(2-fluoro-6-methoxyphenyl)-5*H*-benzo[*c*]pyrimido[4,5-*e*]azepin-2-yl)amino)-2-methoxybenzamido)octyl)amino)-2-oxoethoxy)phenyl)-3-(3,4-dimethoxyphenyl)propyl (*S*)-1-((*S*)-2-(3,4,5-trimethoxyphenyl)butanoyl)piperidine-2-carboxylate (JWJ-01-344)

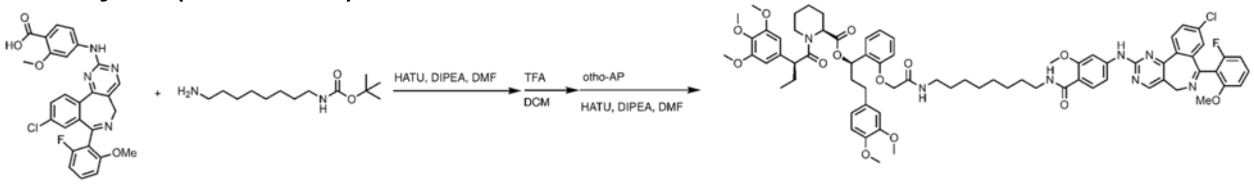

Alisertib JWJ-01-344

By employment of the similar procedure to JWJ-01-342, from alisertib (15 mg, 0.029 mmol), the title compound was prepared (11.8 mg, 27%) with 98% purity. ^{1}H NMR (500 MHz, DMSO-d_6) δ 10.18 (s, 1H), 8.71 (s, 1H), 8.30 (d, *J* = 8.5 Hz, 1H), 8.01 (t, *J* = 5.7 Hz, 1H), 7.95 (s, 1H), 7.84–7.78 (m, 2H), 7.67 (t, *J* = 5.8 Hz, 1H), 7.45–7.38 (m, 2H), 7.19 (tt, *J* = 9.8, 7.1 Hz, 2H), 6.87 (d, *J* = 8.3 Hz, 1H), 6.83–6.76 (m, 3H), 6.73 (d, *J* = 2.0 Hz, 1H), 6.69–6.59 (m, 2H), 6.54 (s, 2H), 6.02 (dd, *J* = 8.3, 4.9 Hz, 1H), 5.32 (dd, *J* = 5.9, 2.4 Hz, 1H), 5.02–4.74 (m, 1H), 4.49 (q, *J* = 14.7 Hz, 2H), 4.04 (d, *J* = 13.1 Hz, 1H), 3.90 (d, *J* = 2.3 Hz, 3H), 3.86 (d, *J* = 7.1 Hz, 2H), 3.70 (s, 6H), 3.68 (s, 4H), 3.63 (s, 1H), 3.54 (s, 7H), 3.25 (q, *J* = 6.7 Hz, 2H), 3.13–3.02 (m, 2H), 2.58 (dd, *J* = 14.0, 10.8 Hz, 1H), 2.47 (d, *J* = 5.0 Hz, 1H), 2.43–2.35 (m, 1H), 2.14 (d, *J* = 13.6 Hz, 1H), 2.05–1.84 (m, 3H), 1.65–1.43 (m, 6H), 1.43–1.31 (m, 3H), 1.20 (d, *J* = 25.4 Hz, 10H), 0.76 (dt, *J* = 34.4, 7.3 Hz, 3H). MS (ESI) *m/z* 661.2 (M + 2H)$^{2+}$/2.

(*R*)-3-(3,4-Dimethoxyphenyl)-1-(2-((1-(4'-((*S*)-6-(2-methoxy-2-oxoethyl)-2,3,9-trimethyl-6*H*-thieno[3,2-*f*][1,2,4]triazolo[4,3-*a*][1,4]diazepin-4-yl)-[1,1'-biphenyl]-4-yl)-1,15-dioxo-5,8,11-trioxa-2,14-diazahexadecan-16-yl)oxy)phenyl)propyl (*S*)-1-((*S*)-2-(3,4,5-trimethoxyphenyl)butanoyl)piperidine-2-carboxylate (JWJ-01-353)

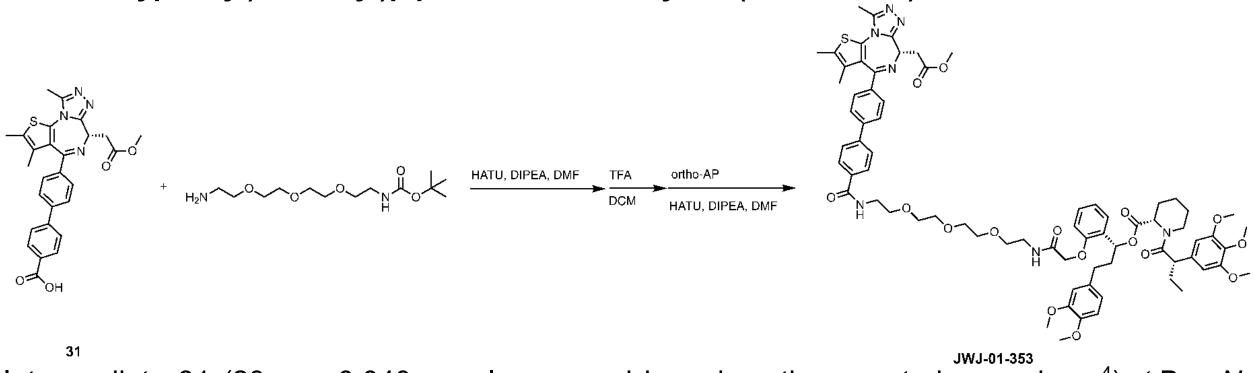

31 JWJ-01-353

Intermediate 31 (20 mg, 0.040 mmol, prepared based on the reported procedures[4]), *t*-Boc-*N*-amido-PEG$_3$-amine (13 mg, 0.044 mmol), HATU (23 mg, 0.060 mmol), and DIPEA (0.035 mL, 0.20 mmol) were charged into an 8 mL scintillation vial. After the addition of DMF (2 mL), the mixture was stirred for 12 hours. Upon the consumption of the alisertib, the mixture was partitioned

between EA (25 mL) and brine (25 mL). The layers were separated in a separator funnel, and the aqueous phase was further extracted with EA (25 mL). The organic extracts were dried over $MgSO_4$, filtered, and concentrated to dryness. The residue was purified by silica gel chromatography to yield the desired intermediate, which was treated with a 1:1 (v/v) mixture of DCM/TFA (5 mL/mmol) for 30 min. Upon the completion of the removal of the Boc group indicated by UPLC, excess TFA and DCM were evaporated under reduced pressure. After putting on the high vacuum for additional 2 hours to fully remove TFA, the residue was dissolved in DMF (1 mL). HATU (1.5 equiv), DIPEA (2 equiv), and the ortho-AP acid (1 equiv) were added to the solution and stirred overnight. Upon the completion of the HATU coupling indicated by UPLC, the mixture was diluted with DMSO to 5 mL and filtered through a 13 mm syringe filter (0.45 um, PTFE). The resulting filtrate was loaded and purified via the preparative HPLC, which yielded the title compound (4.0 mg, 11%) with 96% purity. ^{1}H NMR (500 MHz, DMSO-*d*$_6$) δ 8.59 (t, *J* = 5.7 Hz, 1H), 7.95 (d, *J* = 8.1 Hz, 2H), 7.79 (dd, *J* = 8.5, 2.4 Hz, 4H), 7.52 (d, *J* = 7.9 Hz, 2H), 7.19 (ddd, *J* = 8.9, 6.2, 3.0 Hz, 1H), 6.86 (d, *J* = 8.3 Hz, 1H), 6.82–6.76 (m, 3H), 6.74 (d, *J* = 1.9 Hz, 1H), 6.65–6.60 (m, 1H), 6.55 (s, 2H), 6.02 (dd, *J* = 8.3, 4.8 Hz, 1H), 5.32 (d, *J* = 5.4 Hz, 1H), 4.58–4.48 (m, 3H), 4.04 (d, *J* = 13.3 Hz, 1H), 3.86 (t, *J* = 7.3 Hz, 1H), 3.74 (s, 2H), 3.70 (s, 4H), 3.68 (s, 6H), 3.54 (d, *J* = 7.8 Hz, 8H), 3.53–3.47 (m, 8H), 3.46–3.41 (m, 6H), 3.39 (t, *J* = 5.9 Hz, 2H), 3.25 (hept, *J* = 7.4 Hz, 2H), 2.62 (s, 4H), 2.42 (s, 4H), 2.15 (d, *J* = 13.4 Hz, 1H), 2.05–1.84 (m, 3H), 1.67 (s, 3H), 1.64–1.51 (m, 4H), 1.42–0.96 (m, 2H), 0.76 (dt, *J* = 37.1, 7.3 Hz, 3H). ^{13}C NMR (100 MHz, DMSO-*d*$_6$) δ 172.53, 171.67, 170.96, 168.09, 166.30, 164.52, 154.59, 153.37, 153.19, 149.11, 147.50, 142.06, 141.52, 137.78, 136.46, 136.11, 134.18, 133.84, 132.65, 131.35, 130.64, 130.42, 129.62, 129.35, 129.17, 128.46, 127.31, 127.11, 126.51, 121.66, 120.46, 112.62, 112.55, 112.27, 105.53, 105.30, 70.37, 70.26, 70.21, 70.13, 70.08, 69.43, 69.34, 67.64, 60.52, 60.28, 56.40, 55.98, 55.96, 55.78, 53.97, 52.13, 51.95, 49.22, 43.42, 38.79, 36.93, 36.79, 31.08, 28.62, 26.81, 25.48, 21.03, 14.64, 13.22, 12.89, 12.81, 11.83. MS (ESI) *m/z* 676.0 $(M + 2H)^{2+}/2$. HRMS (m/z): $[M+H]^+$ calcd. for $C_{73}H_{88}N_7O_{16}S$, 1350.6003; found, 1350.6009.

(*R*)-3-(3,4-Dimethoxyphenyl)-1-(2-((1-(4'-((*S*)-6-(2-methoxy-2-oxoethyl)-2,3,9-trimethyl-6*H*-thieno[3,2-*f*][1,2,4]triazolo[4,3-*a*][1,4]diazepin-4-yl)-[1,1'-biphenyl]-4-yl)-1,12-dioxo-5,8-dioxa-2,11-diazatridecan-13-yl)oxy)phenyl)propyl (*S*)-1-((*S*)-2-(3,4,5-trimethoxyphenyl)butanoyl)piperidine-2-carboxylate (JWJ-10-359)

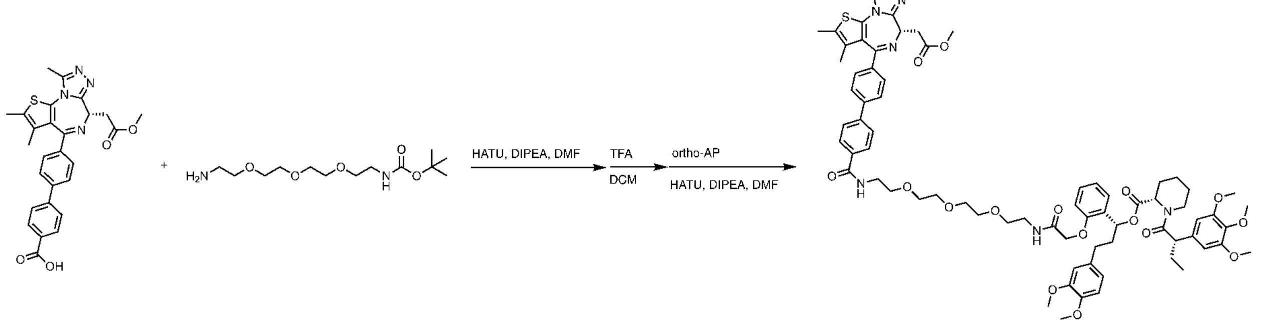

31

JWJ-01-353

By employment of the similar procedure to JWJ-01-353, from intermediate 31 (20 mg, 0.029 mmol), the title compound was prepared (1.7 mg, 5%) with 99% purity. ^{1}H NMR (400 MHz, DMSO-*d*$_6$) δ 8.60 (q, *J* = 5.1 Hz, 1H), 7.98–7.92 (m, 2H), 7.86 (q, *J* = 5.5 Hz, 1H), 7.82–7.75 (m, 4H), 7.51 (dd, *J* = 8.1, 3.0 Hz, 2H), 7.17 (ddd, *J* = 15.4, 8.5, 3.3 Hz, 1H), 6.87–6.72 (m, 4H), 6.64–6.59 (m, 1H), 6.54 (d, *J* = 3.2 Hz, 2H), 6.01 (dt, *J* = 8.2, 4.0 Hz, 1H), 5.32 (d, *J* = 5.0 Hz, 1H), 4.51 (dt, *J* = 9.6, 3.0 Hz, 4H), 4.02 (s, 2H), 3.73 (d, *J* = 3.2 Hz, 2H), 3.70 (d, *J* = 3.2 Hz, 3H), 3.68 (d, *J* = 2.8 Hz, 6H), 3.62 (dd, *J* = 7.0, 3.1 Hz, 2H), 3.54 (d, *J* = 3.2 Hz, 4H), 3.53 (d, *J* = 3.1 Hz, 4H), 3.50 (d, *J* = 3.0 Hz, 2H), 3.47 (s, 2H), 3.44–3.38 (m, 4H), 3.30–3.23 (m, 2H), 2.61 (s, 2H), 2.54 (d, *J* =

3.3 Hz, 5H), 2.43 (d, J = 3.0 Hz, 3H), 1.92 (t, J = 20.2 Hz, 2H), 1.67 (d, J = 3.0 Hz, 3H), 1.61–1.52 (m, 4H), 1.29–1.00 (m, 4H), 0.76 (dtd, J = 30.4, 7.3, 3.1 Hz, 3H). MS (ESI) *m/z* 654.0 $(M + 2H)^{2+}/2$.

SUPPLEMENTARY REFERENCES

1. Li, L., Qiu, D., Shi, J. & Li, Y. Vicinal Diamination of Arenes with Domino Aryne Precursors. *Org. Lett.* **18**, 3726–3729 (2016).
2. Kim, C. H. *et al.* Synthesis of Bispecific Antibodies using Genetically Encoded Unnatural Amino Acids. *J. Am. Chem. Soc.* **134**, 9918–9921 (2012).
3. Sells, T. B. *et al.* MLN8054 and Alisertib (MLN8237): Discovery of Selective Oral Aurora A Inhibitors. *ACS Med. Chem. Lett.* **6**, 630–634 (2015).
4. Hsia, O. *et al. Targeted protein degradation via intramolecular bivalent glues*. Preprint at https://doi.org/10.1101/2023.02.14.528511 (2023).
