# Supplementary material for: A biotin targeting chimera (BioTAC) system to map small molecule interactomes in situ
Source: Nat Commun. 2023 Dec 4;14:8016. doi: 10.1038/s41467-023-43507-5 (PMC10695998; doi:10.1038/s41467-023-43507-5)
Supplement: Supplementary file 3 — Description of Additional Supplementary Files [file 41467_2023_43507_MOESM3_ESM.pdf]

Description of Additional Supplementary Files

File name: **Supplementary Data 1**

Description: Sequence of miniTurbo-FKBP12^{F36V} construct, table of PASEF windows, and complete datasets of proteomic experiments.
