# Supplementary material for: A biotin targeting chimera (BioTAC) system to map small molecule interactomes in situ
Source: Nat Commun. 2023 Dec 4;14:8016. doi: 10.1038/s41467-023-43507-5 (PMC10695998; doi:10.1038/s41467-023-43507-5)
Supplement: Supplementary file 5 — Reporting Summary [file 41467_2023_43507_MOESM5_ESM.pdf]

nature portfolio

Corresponding author(s): Fleur Ferguson, Justin English

Last updated by author(s): Oct 10, 2023

Reporting Summary

Nature Portfolio wishes to improve the reproducibility of the work that we publish. This form provides structure for consistency and transparency in reporting. For further information on Nature Portfolio policies, see our Editorial Policies and the Editorial Policy Checklist.

Statistics

For all statistical analyses, confirm that the following items are present in the figure legend, table legend, main text, or Methods section.

n/a	Confirmed	
☐	☒	The exact sample size (*n*) for each experimental group/condition, given as a discrete number and unit of measurement
☐	☒	A statement on whether measurements were taken from distinct samples or whether the same sample was measured repeatedly
☐	☒	The statistical test(s) used AND whether they are one- or two-sided<br>*Only common tests should be described solely by name; describe more complex techniques in the Methods section.*
☒	☐	A description of all covariates tested
☐	☒	A description of any assumptions or corrections, such as tests of normality and adjustment for multiple comparisons
☐	☒	A full description of the statistical parameters including central tendency (e.g. means) or other basic estimates (e.g. regression coefficient) AND variation (e.g. standard deviation) or associated estimates of uncertainty (e.g. confidence intervals)
☐	☒	For null hypothesis testing, the test statistic (e.g. *F*, *t*, *r*) with confidence intervals, effect sizes, degrees of freedom and *P* value noted<br>*Give P values as exact values whenever suitable.*
☒	☐	For Bayesian analysis, information on the choice of priors and Markov chain Monte Carlo settings
☒	☐	For hierarchical and complex designs, identification of the appropriate level for tests and full reporting of outcomes
☒	☐	Estimates of effect sizes (e.g. Cohen's *d*, Pearson's *r*), indicating how they were calculated

Our web collection on statistics for biologists contains articles on many of the points above.

Software and code

Policy information about availability of computer code

Data collection	ChemiDoc (Bio-Rad), ClarioSTAR Plus (BMG Labtech), Leica SP8 (Leica), Orbitrap Fusion Lumos (Thermo), timsTOF Pro 2 (Bruker)
Data analysis	Image Lab 6.1.0 (Bio-Rad), GraphPad Prism 10.0.0 (GraphPad Software), FIJI 1.54f, PEAKS Studio 10.6 (Bioinformatics Solutions), Protigy 1.1.5 (Broad Institute), DIA-NN 1.8.1

For manuscripts utilizing custom algorithms or software that are central to the research but not yet described in published literature, software must be made available to editors and reviewers. We strongly encourage code deposition in a community repository (e.g. GitHub). See the Nature Portfolio guidelines for submitting code & software for further information.

Data

Policy information about availability of data

All manuscripts must include a data availability statement. This statement should provide the following information, where applicable:

- Accession codes, unique identifiers, or web links for publicly available datasets
- A description of any restrictions on data availability
- For clinical datasets or third party data, please ensure that the statement adheres to our policy

The pCDNA3.1(+) miniTurboID-6xGGSG linker-FKBPF36V-2xHA plasmid has been deposited with Addgene under plasmid # 200641 [https://www.addgene.org/200641/]. The proteomic data generated in this study have been deposited in the PRIDE database under accession code PXD041401 [https://www.ebi.ac.uk/pride/archive/projects/PXD041401] and are available in Supplementary Dataset 1. Source data are provided with this paper.

Research involving human participants, their data, or biological material

Policy information about studies with human participants or human data. See also policy information about sex, gender (identity/presentation), and sexual orientation and race, ethnicity and racism.

Reporting on sex and gender	N/A
Reporting on race, ethnicity, or other socially relevant groupings	N/A
Population characteristics	N/A
Recruitment	N/A
Ethics oversight	N/A

Note that full information on the approval of the study protocol must also be provided in the manuscript.

Field-specific reporting

Please select the one below that is the best fit for your research. If you are not sure, read the appropriate sections before making your selection.

☒ Life sciences ☐ Behavioural & social sciences ☐ Ecological, evolutionary & environmental sciences

For a reference copy of the document with all sections, see nature.com/documents/nr-reporting-summary-flat.pdf

Life sciences study design

All studies must disclose on these points even when the disclosure is negative.

Sample size	Samples sizes were not predetermined using statistical analyses. Sample sizes were selected based on previous experience for each experiment and convention in the field. Information on the number of replicates and independent experiments that were performed for each measurement are disclosed in the manuscript.
Data exclusions	Data were not excluded from analysis.
Replication	Information on the number of replicates and independent experiments that were performed for each measurement are disclosed in the manuscript. Unless otherwise specified, biological experiments were successfully reproduced in n = 3 biological replicates. Quantitative proteomic experiments were performed using n = 3 biological replicates. Attempts at replication were successful.
Randomization	Experiments were not randomized. Randomization was not applicable to this study as cell lines were treated and assessed in the same manner with the appropriate controls.
Blinding	Experiments were not blinded. Blinding was not applicable to this study as data collection or analysis were not prone to bias. All experiments were precise as well as quantitative when possible and were not based on subjective assessments.

Reporting for specific materials, systems and methods

We require information from authors about some types of materials, experimental systems and methods used in many studies. Here, indicate whether each material, system or method listed is relevant to your study. If you are not sure if a list item applies to your research, read the appropriate section before selecting a response.

Materials & experimental systems

n/a	Involved in the study	
☐	☒	Antibodies
☐	☒	Eukaryotic cell lines
☒	☐	Palaeontology and archaeology
☒	☐	Animals and other organisms
☒	☐	Clinical data
☒	☐	Dual use research of concern
☒	☐	Plants

Methods

n/a	Involved in the study	
☒	☐	ChIP-seq
☒	☐	Flow cytometry
☒	☐	MRI-based neuroimaging

Antibodies

Antibodies used	The following primary antibodies were employed in this study: BRD4 (Bethyl Laboratories, A301-985A-M), MEK1/2 (Cell Signaling Technology, 9122S), KSR1 (Cell Signaling Technology, 4640S), DYKDDDDK Tag (Cel Signaling Technology, 14793S), alpha-tubulin (Cell Signaling Technology, 3873S), Aurora A (Cell Signaling Technology, 14475T), HA-tag (Cell Signaling Technology, 3724S), BRD4 (Cell Signaling Technology, 63759S), MEK1/2 (Cell Signaling Technology, 4694S). Species-specific fluorescently labeled infrared secondary antibodies including: DyLight 680 anti-mouse IgG (Cell Signaling Technology, 5470s), DyLight 800 anti-rabbit IgG (Cell Signaling Technology, 5151S), Alexa Fluor 488 AffiniPure Donkey Anti-Rabbit (Jackson ImmunoResearch, 711-545-152), and Alexa Fluor 647 AffiniPure Donkey Anti-Mouse (Jackson ImmunoResearch, 715-605-151) were employed as appropriate.
Validation	All antibodies employed in this study for immunoblotting are commercially available and were validated by the manufacturer as follows: BRD4 (Bethyl Laboratories, A301-985A-M): the vendor shows detection of BRD4 by immunoblotting in multiple cell lines. MEK1/2 (Cell Signaling Technology, 9122S): the vendor shows detection of MEK1/2 by immunoblotting and cross-validation with a second anti-MEK antibody. KSR1 (Cell Signaling Technology, 4640S): the vendor shows detection of KSR1 by immunoblotting in multiple cell lines, and shows specificity via lack of detection in non-hKSR1 expressing lines. DYKDDDDK Tag (Cell Signaling Technology, 14793S): the vendor shows detection of divergent, exogenously expressed DYDDDDK-tagged fusions by immunoblotting, including non-tagged controls. alpha-tubulin (Cell Siganling Technology, 3873S): the vendor shows detection by immunoblotting in multiple cell lines. Aurora A (Cell Signaling Technology, 14475T): the vendor shows detection of Aurora A by immunoblotting non-synchronized and synchronized cells. HA-tag (Cell Signaling Technology, 3724S): the vendor shows detection of divergent, exogenously expressed HA-tagged fusions by immunoblotting, including non-tagged controls. BRD4 (Cell Signaling Technology, 63759S): the vendor shows detection of BRD4 by immunoblotting in multiple cell lines. MEK1/2 (Cell Signaling Technology, 4694S): the vendor shows detection of MEK1/2 by immunoblotting in multiple cell lines.

Eukaryotic cell lines

Policy information about cell lines and Sex and Gender in Research

Cell line source(s)	The following cell lines were employed in this study: HEK293 (ATCC, CRL-1573), A549 (ATCC, CCL-185), HCT116 (ATCC, CCL-247), HEK293FT-FKBP12F36VNluc (N. S. Gray lab).
Authentication	Authentication was performed by standard methods which include STR profiling.
Mycoplasma contamination	All cell lines tested negative for mycoplasma.
Commonly misidentified lines (See ICLAC register)	No commonly misidentified lines were used in this study.
